# Phosphorus Acquisition Strategies Among Phytoplankton and Free‐Living Bacterial Communities in the Baltic Proper

**DOI:** 10.1111/1758-2229.70332

**Published:** 2026-04-16

**Authors:** Mollica Thomas, Farnelid Hanna, Lindehoff Elin, Lundin Daniel, Pinhassi Jarone, Legrand Catherine

**Affiliations:** ^1^ Centre for Ecology and Evolution in Microbial Model Systems—EEMiS Linnaeus University Kalmar Sweden; ^2^ School of Health and Welfare Jönköping University Jönköping Sweden

**Keywords:** bacterioplankton, Baltic Sea, metatranscriptomics, phosphorus, phytoplankton, remodelling, transporters

## Abstract

Nutrient limitation in the Baltic Proper exhibits temporal variations, with nitrogen limiting diatom and dinoflagellate‐dominated spring blooms, while phosphorus constraints characterise the cyanobacterial summer blooms. Phosphorus is a key element for cellular functions and poses significant challenges for planktonic microbial communities under limited availability. Numerous studies have explored strategies phytoplankton and bacteria employ to cope with phosphorus scarcity. However, the temporal dynamics of phosphorus acquisition within natural communities remain poorly understood. Using metatranscriptomics, this study addresses this gap by examining how planktonic microbial communities acquire phosphorus over a year‐long monitoring at an offshore station. Targeting genes related to phosphorus degradation, transport and membrane remodelling, we unveil diverse strategies employed by planktonic microbial communities to acquire phosphorus. Our findings highlight transporter and membrane remodelling‐related genes are expressed at high levels across the year, suggesting their important role in coping with phosphorus acquisition. Our dataset reveals distinct strategies between phytoplankton and free‐living bacteria under nutrient‐limited conditions. While eukaryotic phytoplankton appear to rely more on recycling internal stores of phosphorus via membrane remodelling processes, free‐living bacteria appear more prone to optimize extracellular scavenging mechanisms. These insights reveal the complex physiological adjustments of marine microbial communities to fluctuating phosphorus availability in the Baltic Sea.

## Graphical abstract

Phosphorus is a key element for cellular functions and poses significant challenges for planktonic microbial communities under limited availability. Metagenomics highlighted that heterotrophic bacteria predominantly relied on phosphate scavenging while eukaryotic phytoplankton primarily utilized membrane remodelling to cope with limited phosphorus conditions.

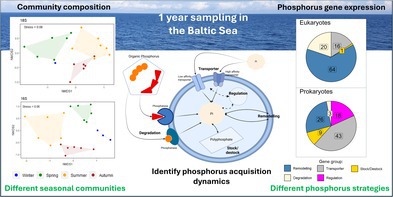

## 1 Introduction

Phosphorus (P), a key cellular component found in DNA, membrane lipids and nucleotides exists in marine environments both as bioavailable inorganic phosphate and within complex dissolved organic compounds such as phosphonate. While P is less frequently the limiting nutrient in marine systems compared to nitrogen, it is crucial—for example, in oligotrophic areas (Moore et al. 2013)—and the study of P uptake has gained attention in the context of eutrophication, where excess P can lead to harmful algal blooms and hypoxic conditions. The Baltic Sea has experienced significant eutrophication since the 20th century, with P levels peaking in the 1980s (The BACC II Author Team 2015). Despite mitigation efforts, a large part of the Baltic Sea remained affected by eutrophication with no clear recovery patterns between 2016 and 2021 compared to 2011–2016 (HELCOM 2023). Elevated nutrient levels stimulate phytoplankton blooms, increasing organic matter deposition. Mineralization of this organic matter at the seafloor depletes bottom water oxygen levels, leading to hypoxia (< 2 mg L^−1^ dissolved oxygen, Kuliński et al. 2022). Hypoxic areas in the Baltic Sea increased by 7% and anoxic (0 mg L^−1^ dissolved oxygen) zones by 11% between the periods 1960–1998 and 1999–2021 (SMHI data, Hansson and Viktorsson 2023). Under such low bottom water oxygen conditions, P bound to Fe‐oxides in sediments is released back into the water column (Emeis et al. 2000; Mort et al. 2010), contributing to further P enrichment sustaining a feedback loop known as the ‘vicious cycle of phosphorus’ (Vahtera et al. 2007).

The Baltic Proper is the largest water body of the Baltic Sea, and its primary production cycle includes a nitrogen‐limited spring bloom dominated by diatoms and dinoflagellates, and a P‐limited summer bloom dominated by diazotrophic cyanobacteria and dinoflagellates (Degerholm et al. 2006; Nausch and Nausch 2006; Walve and Larsson 2007; Legrand et al. 2015). Bacterial biomass follows a seasonal trend, peaking with highest abundances in summer (Fridolfsson et al. 2023). Different components of the prokaryotic microbial communities peak at different times: Bacteroidetes in spring, Cyanobacteria and Verrucomicrobia in summer and Actinobacteria in autumn (Lindh et al. 2015; Fridolfsson et al. 2023). An increased dissolved inorganic phosphorus (DIP) flux from sediments supports greater blooms of diazotrophic cyanobacteria during summer, increasing fixed nitrogen, affecting the length of the spring bloom and annual sedimentation (Lignell et al. 1993; Heiskanen and Kononen 1994; Vahtera et al. 2007; Viktorsson et al. 2013). However, it is still unclear how elevated nutrient levels, when combined with seasonal changes, influence nutrient uptake and storage mechanisms in planktonic communities.

Planktonic communities show diverse P requirements and can use a range of uptake strategies, which can differ significantly at both genus and species levels. To adapt to low P availability, phytoplankton and bacteria utilize multiple mechanisms, including adjusting their affinity for DIP, storing excess P, reallocating internal reserves, or enzymatically liberating P from dissolved organic phosphorus (DOP). A key adaptation among these strategies involves the role of transporters in regulating DIP acquisition. Organisms express either high‐ or low‐affinity transporters based on environmental conditions. High‐affinity transporters such as the pstSCAB protein are predominantly observed in bacteria such as *Prochlorococcus* and become more active under P‐limited conditions (Martiny et al. 2006; Gardner and McCleary 2019). Conversely, low‐affinity P transporters like PiT are expressed when P is more abundant and are found across various plankton families (Lin et al. 2016). Analogous high and low‐affinity P transport systems have been identified in Dinophyta (Lin et al. 2016), Haptophyta (Beszteri et al. 2012), Chlorophyta (Moseley et al. 2006) and in picoeukaryotes (Hagström et al. 2021). Consequently, the relative expression patterns of these transport systems can give valuable insights into ambient P conditions where the expression of high‐affinity transporters signal limited resources while low‐affinity counterparts indicate abundance.

Another strategy to manage P limitation is the recycling of internal P reserves. Polyphosphate, nucleic acids and membrane lipids constitute the main intracellular pool of P, with RNA being the predominant fraction (Geider and La Roche 2002; Kwiatkowski et al. 2018). Polyphosphate serves as a significant P reservoir in all kingdoms (Kornberg et al. 1999; Solovchenko et al. 2019; Sanz‐Luque et al. 2020). In prokaryotes and some eukaryotes, polyphosphate synthesis is facilitated by the key enzyme polyphosphate kinase (*PPK*) (Brown and Kornberg 2004, 2008). Most eukaryotes, however, rely on vacuole transporter chaperones such as VTC 1–4 for this process (Ogawa et al. 2000; Dyhrman et al. 2012). Degradation of polyphosphate involves exophosphatase (*PPX*), which exists in both prokaryotic and eukaryotic cells (Kornberg et al. 1999). Additionally, lipid remodelling replacing phospholipids with non‐phosphorus‐based lipids, is an adaptation observed in some phytoplankton under low P conditions (Van Mooy et al. 2009; Martin et al. 2011; Cañavate et al. 2017), though the mechanisms driving this variability among taxa remain unresolved (Mühlroth et al. 2017). While lipid remodelling was once thought be exclusive to phytoplankton and absent in heterotrophic bacteria (Van Mooy et al. 2009), research identified glycolipids within SAR11 membranes (alphaproteobacteria) in the oligotrophic Sargasso Sea (Carini et al. 2015) and highlighted phospholipase c (*PlcP*) as a key gene for lipid remodelling in these bacteria (Sebastián et al. 2016). However, understanding of lipid remodelling in heterotrophic bacteria under P limitation remains incomplete.

The significance of DOP in surface waters has gained considerable attention, particularly in relation to how planktonic microbial communities adapt their strategies for utilizing and recycling P when it is scarce (Ruttenberg and Dyhrman 2005; Dyhrman et al. 2007). When P is limited, the ability to access it from DOP is a critical advantage for survival. Among key compounds in DOP are phosphonates, which can be used as alternative P sources when degraded by phosphonases. While the degradation of phosphonate has been well documented in heterotrophic bacteria and cyanobacteria, its occurrence in eukaryotic phytoplankton remains unreported (Lin et al. 2016). Genes associated with phosphonate degradation (*phn*) are prevalent in filamentous cyanobacteria, enabling utilization of methylphosphonate as the sole source of P (Zhao et al. 2022). Phosphatases, essential for the breakdown of DOP, are categorized in alkaline and acidic types and have been extensively studied in prokaryotes (Theodorou et al. 1991; Lubián et al. 1992; Lin et al. 2016), particularly through their association with the pho operon (reviewed in Gardner and McCleary 2019). These enzymes have also been identified in major groups of eukaryotic phytoplankton groups including diatoms (Dyhrman et al. 2012) and dinoflagellates (Lin et al. 2012). The identification of phosphatases in model eukaryotic species are a starting point for understanding how natural communities may respond when inorganic P‐pools become depleted.

As eutrophication and climate change intensify, P is expected to play an increasingly crucial role in shaping primary production in the Baltic Sea, notably with the cyanobacterial summer blooms. However, the complexities of P cycling are still being studied to understand how increased P levels will affect phytoplankton and bacterial communities, and how these changes, in turn, influence ecosystem functions. Cyanobacteria show adaptability to varying P conditions by using a wide range of bioavailable P sources (see review by Xiao et al. 2022). For example, beyond sedimentary fluxes of P from deoxygenated environments, several hypotheses address the P sources fuelling diazotrophic cyanobacterial blooms during summer in the Baltic Sea Proper. Some studies suggest internal reserves like ATP or nucleotides are preferred for growth maintenance (Nausch and Nausch 2004; Nausch et al. 2009; Walve and Larsson 2010), others highlight DOP utilization as critical (Dyhrman et al. 2007; Nausch et al. 2018). In the Eastern Gotland Basin, DOP components like ATP, DNA and phospholipids account for approximately 2.4%–5.4% of DOP (Nausch et al. 2018). Despite their relatively low abundance within DOP pools, these labile compounds are thought to serve as significant P sources for phyto‐ and bacterioplankton in the Baltic Sea (Nausch and Nausch 2004, 2006; Nausch et al. 2008, 2018). To elucidate these hypotheses further, investigating the response of functional plankton groups and community level interactions with P in their natural environment becomes critical. Metatranscriptomic approaches paired with traditional methodologies offer potential for understanding how phyto‐ and bacterioplankton respond to changing P levels at a cellular level by revealing detailed patterns of gene expression.

This study involved a comprehensive year‐long sampling at the Linnaeus Microbial Observatory (LMO), an offshore station in the Baltic Proper, with the aim of examining P acquisition strategies within pelagic microbial communities. Using metatranscriptomics, we explored the mechanisms of P acquisition employed by both eukaryotic phytoplankton and free‐living prokaryotic communities. Gaining a deeper understanding of metabolic P strategies under natural conditions is crucial for uncovering how planktonic microbial communities adapt to and respond to environmental changes.

## 2 Material and Methods

### 2.1 Sampling

Water was sampled at the Linnaeus Microbial Observatory (LMO, N 56° 55.8540′, E 17° 3.6420′) from February 2020 to March 2021 on 26 occasions. The sampling frequency was initially set to every second week but was occasionally adjusted based on weather conditions. Temperature and salinity were measured with a AAQ 1186‐H CTD (Alec Electronics, Japan), and a 5 L Ruttner sampler was used to collect water at five discrete depths (2, 4, 6, 8 and 10 m). The water samples from the different depths were mixed in a 25 L canister, stored on the ship deck in the shade and brought back to the field laboratory within 30 min.

### 2.2 Nutrient Pools and Chlorophyll a

Water for measuring dissolved inorganic nutrients (phosphate, ammonium, nitrate and nitrite) was collected in an acid washed bottle (500 mL) and stored at −20°C until analysis using standard protocols (UV‐spectrophotometer, Hach DR 3900, Valderrama 1995). Detection limits were as follows: 0.1 μM for ammonia, nitrate and nitrite; 0.03 μM for phosphate (Valderrama 1995). Samples for particulate organic carbon (POC) and nitrogen (PON), and phosphorus (POP) determination were collected by filtration of seawater (500 and 250 mL, respectively) through precombusted (3 h, 475°C) Whatman GF/F filters and dried overnight at 60°C. POC and PON were analysed using standard CHN analysis (Perkins‐Elmer 2400 series II elemental analyser, detection limits of 1 μg for both) and POP was measured according to Solorzano and Sharp (1980/SS‐EN ISO 6878‐2005, detection limit of 5 μg/L) slightly modified according to SS‐EN ISO 6878‐2005. Chlorophyll *a* was measured on a fluorometer (Trilogy Turner Designs) after filtration of 500 mL of water on A/E filters and overnight extraction in 4 mL of 96% ethanol based on Jespersen and Christoffersen (1987).

### 2.3 Biomass and Community Composition

For phytoplankton enumeration using microscopy, 100 mL of water was fixed using acid Lugol (Neutral Lugol with glacial acetic acid at 1% final concentration), kept at 4°C and counted using the Utermöhl (1958) with a Nikon TMS (Tokyo, Japan). Phytoplankton carbon biomass concentration was derived from cell abundance and cellular carbon on phyla level (Olenina et al. 2006, version 2018). Samples for determining bacteria and picophytoplankton cell abundances using flow cytometry were prefiltered (< 3 μm, Whatman GF/D filter precombusted 3 h at 475°C), fixed with grade I glutaraldehyde (1% final concentration) and stored at −80°C until analysis. Heterotrophic bacteria were analysed using a 488 nm argon laser Beckton Dickinson FACSCalibur (Olson et al. 1985), and picophytoplankton using a CyFlow Cube 8 flow cytometer (Partec, Jettingen‐Scheppach, Germany) and identified as described in Alegria Zufia et al. (2021). A carbon conversion factor of 20 fg C cell^−1^ was used for bacterioplankton (Lee and Fuhrman 1987) and a proxy of 243.5 fg C cell^−1^ was applied for picophytoplankton cells (Campbell et al. 1994; Worden et al. 2004).

### 2.4 DNA Extraction, Amplification and Sequencing

DNA samples for community composition analysis were collected by filtering 250–350 mL water onto a 25 mm diameter 0.2 μm Supor filter (Pall Corporation, USA), and stored at −80°C until extraction. Due to the limited water volume available and time needed to filter the water, only one biological replicate was taken for each sampling occasion. DNA was extracted using the FastDNA SPIN Kit for soil from MP Biomedicals Inc. using Lysing matrix Y according to the manufacturer's instructions with an addition of an incubation with proteinase‐K (1% final concentration) at 55°C for 1 h. For amplification of 16S rRNA gene fragments, the V3–V4 region was targeted using primers 341F and 805R (Herlemann et al. 2011). For amplification of the 18S rRNA gene, the V4 region was targeted, using the primers 454F (Stoeck et al. 2010) and V4RB (Balzano et al. 2015). Primer details and PCR protocols are described in Table S1. All PCRs were done using the Phusion High‐Fidelity PCR Master Mix (Thermo‐Fisher). A second PCR was performed to attach i5 and i7 NEXTERA indexes for Illumina sequencing (Table S1). After each PCR, the products were purified using AMPure XP (Beckman Coulter). The products were quantified with a Qubit fluorometer to determine concentrations for pooling of samples. Indexed samples were sequenced using Illumina MiSeq (Illumina Inc., USA) with 2 × 300 cycles paired‐end sequencing at SciLifeLab. On the 26 samplings, a total of 21 samples were successfully sequenced for the 16S and 20 were successful for the 18S.

### 2.5 RNA Extraction, Amplification and Sequencing

RNA samples for transcriptomic analysis of the eukaryotic community were collected by filtering 250–350 mL water onto 25 mm diameter 0.2 μm Supor‐200 filters (Pall Corporation, USA), referred to as ‘unfiltered’. Due to low yields of RNA from the unfiltered samples, the analysis of the prokaryotic gene expression of filamentous cyanobacteria was not possible. RNA samples for transcriptomic analysis of the free‐living prokaryotic community were collected on a 0.2 μm Supor filter, after a pre‐filtration of the water through a 3 μm Whatman GF/D filter, referred to as ‘pre‐filtered.’ All 0.2 μm filters were collected and stored at −80°C until extraction. One biological replicate was taken for both unfiltered and pre‐filtered samples for each sampling occasion.

RNA was extracted using the Lysing matrix E (MP Biomedicals) and RNeasy mini kit (Qiagen Cat No./ID: 74104). RNA quality control was checked using Tapestation (Tapestation 4150, Agilent Technologies, USA) prior to DNase treatment with AMBIONTurbo DNA free kit (Invitrogen). For unfiltered samples (14 samples), library preparation was done by SciLifeLab/NGI (Stockholm, Sweden) after Poly‐A enrichment (Illumina TruSeq Stranded mRNA). Library preparation for the pre‐filtered samples (12 samples) included ribosomal depletion using the RiboMinus Transcriptome Isolation kit (Thermo Fisher) with the RiboMinus Concentration Module. The synthesis of cDNA and sequencing for each sample was done by SciLifeLab/NGI (Stockholm, Sweden). Constructed libraries were sequenced using NovaSeq6000 (NovaSeq Control Software 1.7.5/RTA v3.4.4) at SciLifeLab.

### 2.6 Bioinformatics: Amplicons

Reads for 16S and 18S rRNA gene amplicons were separately screened for sequencing errors using nf‐core/ampliseq (Straub et al. 2020, version 2.3.2) which runs on Nextflow (Ewels et al. 2020, version 22.04.5) and DADA2 (Callahan et al. 2016, version 1.28.0). Taxonomy of the resulting amplicon sequence variants (ASVs) was done using the SBDI Sativa curated Genome Taxonomy Database (GTDB) for 16S (Lundin and Andersson 2021, release R07‐RS207‐1) and The Protist Ribosomal Reference database (PR(2), Guillou et al. 2012, version 5.0.0) for 18S. All details about the number of reads used for the analysis are shown in Table S2.

### 2.7 Assembly, Quantification and Annotation

Metatranscriptomes were processed with the assembly pipeline nf‐core/metatdenovo (Di Leo et al. 2025 version 1.0.0) using Megahit (Li et al. 2015) as assembler and open reading frames (ORFs) were called with Prokka for free‐living prokaryotes (Seemann 2014), and TransDecoder for eukaryotes (Haas, n.d.). For both domains, functional annotation of ORFs was done by the pipeline using KofamScan (Aramaki et al. 2020, version 1.3.0); and both taxonomy was assigned with EUKulele (Krinos et al. 2021, version 2.0.5) using GTDB (Parks et al. 2018, v. R07‐RS207) for free‐living prokaryotic transcripts (pre‐filtered samples) and The Marine Microbial Eukaryote Transcriptome Sequencing Project (Keeling et al. 2014; Johnson et al. 2018, MMETSP version 2) for eukaryotic transcripts (unfiltered samples).

### 2.8 P‐Acquisition Gene Identification

To investigate the P acquisition strategies in the bacterial planktonic community, a list of genes of interest was built based on the KEGG orthologs (KO) identified by KofamScan (Table 1). A total of five gene categories of interest were targeted: degradation, remodelling, stock/destock, transporters (both free‐living prokaryotes and eukaryotes) and regulation (prokaryote, Table 1). Concerning the remodelling of the membrane, identifying relevant KOs in the metabolism of phospholipids was difficult; we decided to choose a large panel of KOs all related to the synthesis of the main phospholipids (Figure S1, Kanehisa et al. 2025).

**TABLE 1:** List of the genes analysed in this study.

Category	ko	Gene name	EC number	Function
Degradation	K19670	phnA		Hydrolase
	K05306	phnX		
	K14379	ACP5		Phosphatase
	K22390	ACP7		
	K01077	pho*		
	K01113	phoD		
	K06162	phnM		
	K09474	phoN		
	K06167	phnP		Phosphodiesterase
	K02043	phnF		Phosphonate transport
	K05781	phnK		
	K03430	phnW		Transaminase
	K06166	phnG		Transferase
	K06164	phnI		
Membrane remodelling	K13621	BTA1		Betaine lipid synthase
	K00006	GPD1	1.1.1.8	Oxidoreductase
	K00111	glpA/glpD	1.1.5.3	
	K00112	glpB	1.1.5.3	
	K00113	glpC		
	K00057	gpsA	1.1.1.94	
	K18693	DPP1, DPPL, PLPP4_5	3.6.1.75; 3.1.3.4	Phosphatase
	K15728	LPIN	3.1.3.4	
	K01080	PLPP1_2_3	3.1.3.4	
	K01004	pcs	2.7.8.24	Phosphatidylcholine synthase
	K08729	PTDSS1	2.7.8.‐	Phosphatidylserine synthase
	K08730	PTDSS2	2.7.8.29	
	K01126	glpQ, ugpQ	3.1.4.46	Phosphodiesterase
	K16818	DAD1	3.1.1.32	Phospholipase
	K22389	LCAT3	3.1.1.32	
	K01047	PLA2G, SPLA2	3.1.1.4	
	K16817	PLA2G16	3.1.1.32; 3.1.1.4	
	K16342	PLA2G4, CPLA2	3.1.1.4	
	K16343	PLA2G6, IPLA2	3.1.1.4	
	K14621	PLB1, PLB	3.1.1.4; 3.1.1.5	
	K01115	PLD1_2	3.1.4.4	
	K16860	PLD3_4	3.1.4.4	
	K22697	SAMD8	3.1.4.62	
	K04714	SGMS	2.7.8.27; 3.1.4.62	
	K14674	TGL4	3.1.1.3; 3.1.1.13; 3.1.1.4; 2.3.1.51	
	K06900	capV	3.1.1.32; 3.1.1.‐	
	K01114	plc	3.1.4.3	
	K17717	pld	3.1.4.4	
	K01058	pldA	3.1.1.32; 3.1.1.4	
	K14286	AGXT2L1, ETNPPL	4.2.3.2	Phospho‐lyase
	K13523	AGPAT3_4	2.3.1.51; 2.3.1.‐	Transferase
	K13509	AGPATa_2	2.3.1.51	
	K00981	CDS1, CDS2, cdsA	2.7.7.41	
	K13644	CEPT1	2.7.8.1; 2.7.8.2	
	K14156	CHK	2.7.1.32; 2.7.1.82	
	K00866	CKI1	2.7.1.32	
	K16368	DGK1	2.7.1.174	
	K00894	ETNK, EKI	2.7.1.82	
	K13507	GAT	2.3.1.15; 2.3.1.42	
	K00649	GNPAT	2.3.1.42	
	K13513	LCLAT1, AGPAT8	2.3.1.‐; 2.3.1.51	
	K22831	LOA1	2.3.1.51	
	K13510	LPCAT1_2	2.3.1.23; 2.3.1.67	
	K13512	LPCAT4, AGPAT7	2.3.1.23; 2.3.1.‐	
	K13519	LPT1, ALE1	2.3.1.51; 2.3.1.23; 2.3.1.‐	
	K13517	MBOAT1_2	2.3.1.51; 2.3.1.‐	
	K05929	NMT	2.1.1.103	
	K00968	PCYT1	2.7.7.15	
	K00967	PCYT2	2.7.7.14	
	K00551	PEMT	2.1.1.17; 2.1.1.71	
	K00550	PLMT	2.1.1.71	
	K06119	SQD2	2.4.1.‐	
	K13622	btaA		
	K13623	btaB		
	K07029	dagK	2.7.1.107	
	K00901	dgkA, DGK	2.7.1.107	
	K00655	plsC	2.3.1.51	
	K00570	pmtA	2.1.1.17; 2.1.1.71	
	K06118	SQD1	3.13.1.1	UDP‐sulfoquinovose synthase
Regulation	K07657	phoB		Signal transduction
	K07658	phoB1, phoP		
	K06217	phoH, phoL		
	K07175	phoH2		
	K07636	phoR		
	K07776	regX3		
	K07768	senX3		
	K02039	phoU		Transporter
Stock/destock	K00937	ppk1		Polyphosphate kinase
	K22468	ppk2		
	K23753	ppk2, pap		
	K01524	ppx‐gppA		Exopolyphosphatase
Transporter	K08176	PHO84		Transporter
	K14430	PHO87_91		
	K03306	PiT*		
	K02041	phnC		
	K02044	phnD		
	K02042	phnE		
	K16322	pit		
	K02038	pstA		
	K02036	pstB		
	K02037	pstC		
	K02040	pstS		

*Note:* EC numbers have been added for membrane remodelling when available to help locating the genes in Figure S1.

Once the P‐related gene list was established, ORFs in the dataset associated with P‐related genes were selected for analysis based on their KO number (Table 1) using the KofamScan table. ORFs with significant matches to multiple KOs were assigned to the one it scored best to. Combining all transcriptomic samples for the free‐living prokaryotic community, a total of 1,776,196 ORFs were detected of which 568,408 ORFs were attributed to Bacteria. After filtering for P‐related genes, a total of 5947 ORFs were identified. For eukaryotic metatranscriptomes, a total of 5,515,270 ORFs were detected and 2,023,388 were annotated and attributed to Eukaryota of which 1,972,809 ORFs were attributed to phytoplankton and a total of 9319 ORFs of P‐related genes were identified.

### 2.9 Statistics and Plotting

Data handling and plotting was done using R (R Core Team 2022) using Tidyverse (Wickham et al. 2019), ggConvexHull (Martin 2017) and cowplot (Wilke 2021). To analyse the amplicon libraries, the phyloseq (McMurdie and Holmes 2013) package was used. Statistical analyses (NMDS, adonis) were done with Vegan (Oksanen et al. 2020) after scaling with ranked subsampling using the SRS package (Beule and Karlovsky 2020). Seasons were defined according to calendar days (Winter: 21/12/2019; Spring 2020: 20/03/2020; Summer: 20/06/2020; Autumn: 22/09/2020; Winter: 21/12/2020). All correlation tables are based on Spearman correlation with a significance level set at *ρ* = 0.05 and plotted using corrplot (Wei and Simko 2021) and pheatmap (Kolde 2018).

## 3 Results

### 3.1 Environmental Context

**FIGURE 1:**
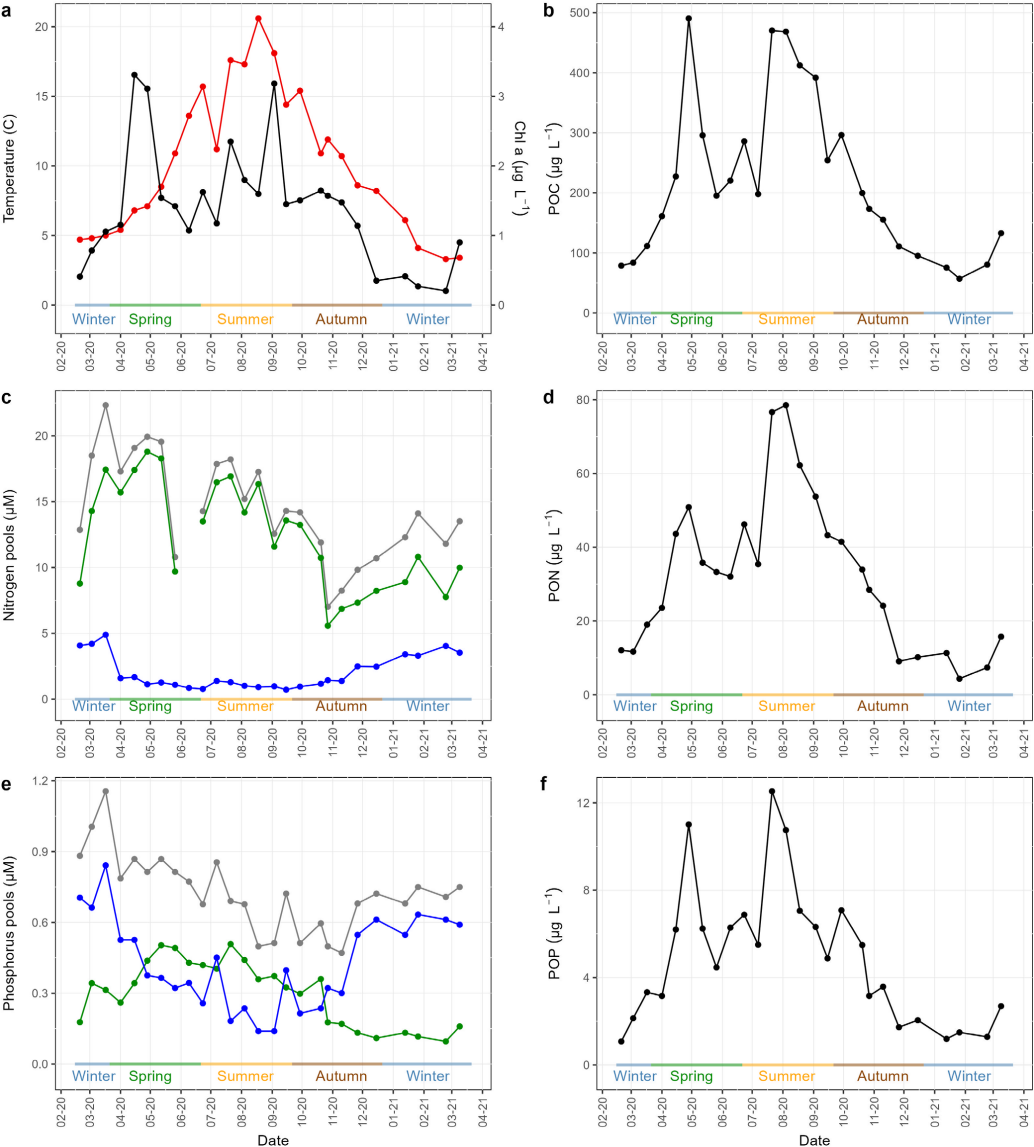
Seasonal variation of (a) temperature (red) and chlorophyll a (black), (b) particulate organic carbon (POC), (c) nitrogen pools with dissolved inorganic nitrogen (blue), dissolved organic nitrogen (green) and total dissolved nitrogen (grey), (d) particulate organic nitrogen (PON), (e) phosphorus pools with dissolved inorganic phosphorus (blue), dissolved organic phosphorus (green) and total dissolved phosphorus (grey), (f) particulate organic phosphorus (POP). Environmental parameters were measured at the Linnaeus Microbial Observatory, Baltic Sea between February 2020 and April 2021.

The temperature variability and nutrient dynamics observed at LMO from February 2020 to March 2021 provide a comprehensive understanding of seasonal changes in this ecosystem (Mollica et al. 2025). During this period, temperature ranged from a low 3.3°C during a relatively mild winter to a high of 20.6°C in summer 2020 (Figure 1a). These variations align with historical data recorded at LMO since 2011 (Legrand et al. 2015; Fridolfsson et al. 2019, 2023). Phytoplankton blooms were characterized by chlorophyll *a* (chl *a*) concentrations reaching maximum values of 3.3 mg m^−3^ in spring and 3.2 mg m^−3^ in summer (Figure 1b). Corresponding peaks in POC, PON and POP were observed along these chl *a* maxima, particularly marked in July but absent during the September bloom (Figure 1d,f). Seasonal changes were also observed in the inorganic nutrient concentrations; DIN peaked at the end of winter (4.9 μM) before decreasing to around 0.8 μM during summer to recover to winter levels (~4.0 μM) by autumn (Figure 1c). DIP followed similar patterns, reaching ~0.8 μM in winter but declining post spring blooms to lows (< 0.3 μM) in late summer and recovery from November on (Figure 1e). DOP had an opposite pattern compared to DIP, increasing from 0.2 μM in winter to 0.5 μM in summer and continuously decreasing through late summer to autumn to reach ~0.1 μM as winter started (Figure 1e).

### 3.2 Biomass and Community Composition

**FIGURE 2:**
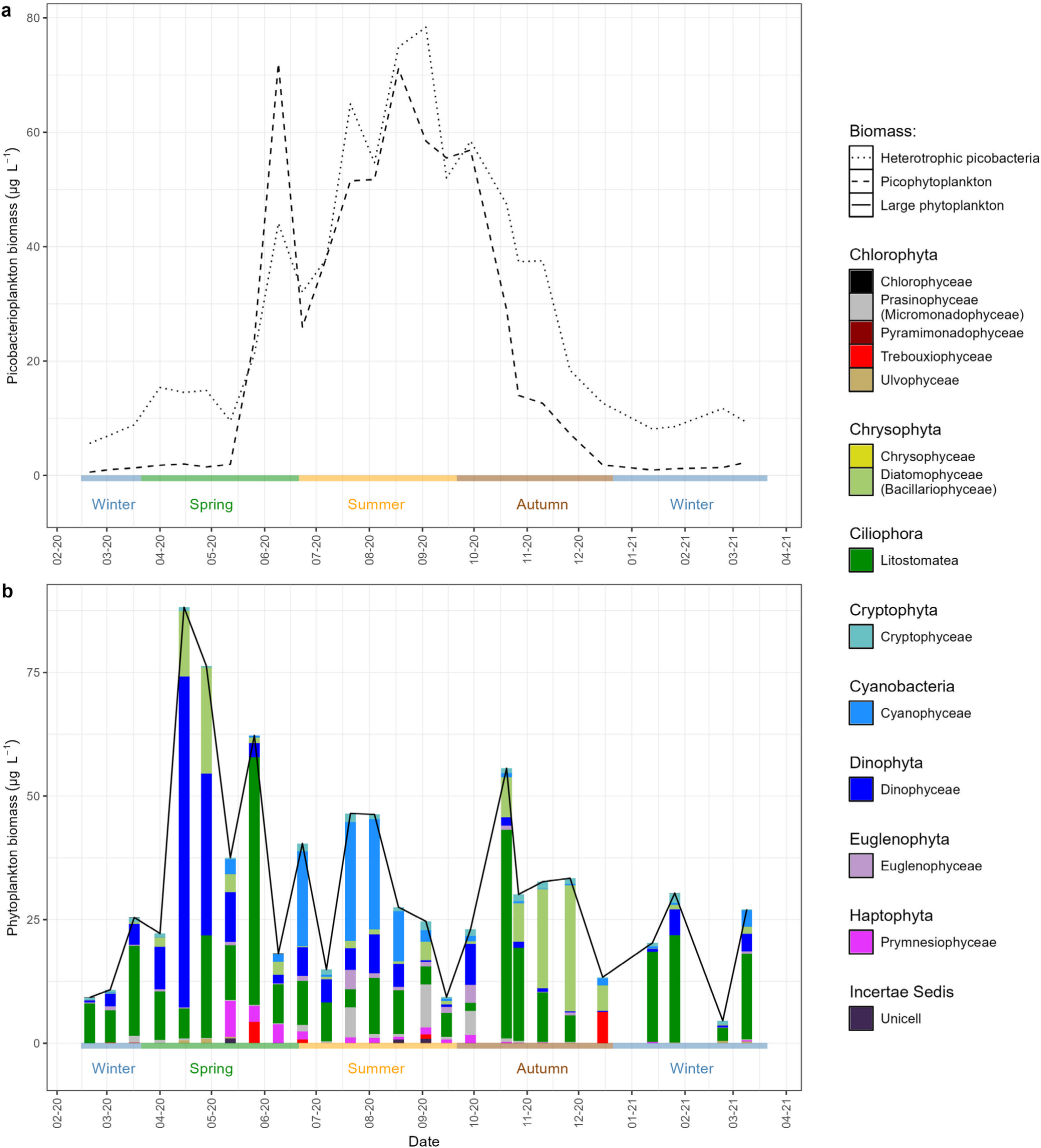
Annual changes in bacterio‐ and phytoplankton carbon per phylum, (a) picoplankton (free‐living prokaryotes and picoeukaryotes) biomass based on flow cytometry, (b) large phytoplankton biomass based on microscopy, total (–) and changes in biomass per phylum.

**FIGURE 3:**
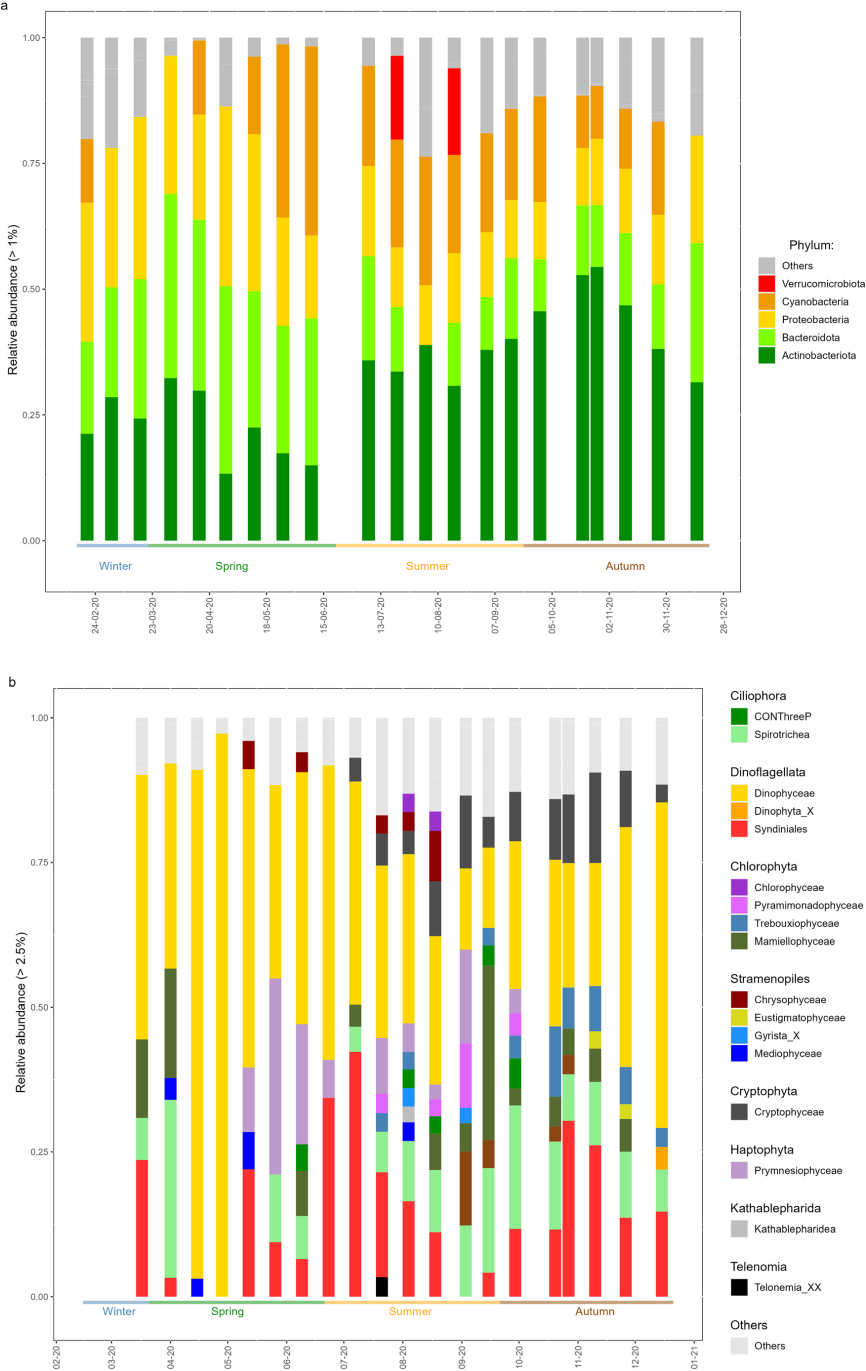
Relative abundance percentages of major (a) bacterial phyla and (b) eukaryotic plankton communities, based on 16S and 18srRNA gene sequences, at the Linnaeus Microbial Observatory, Baltic Sea between February 2020 and April 2021.

**FIGURE 4:**
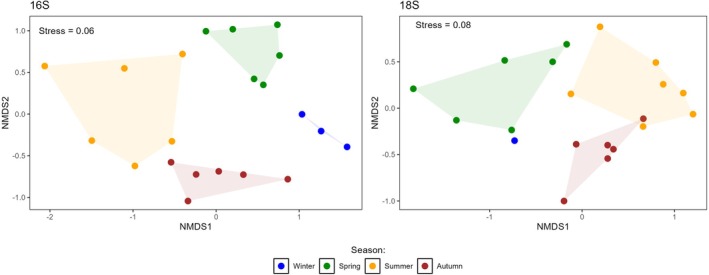
Non‐metric multidimensional scaling (NMDS) ordination of eukaryotic (left) and bacterial communities (right) at the Linnaeus Microbial Observatory, Baltic Sea.

The seasonal dynamics of bacterioplankton biomass and community composition varied markedly, reflecting the environmental changes across seasons. From winter to mid‐spring, bacterioplankton biomass remained relatively low (5–15 mg C m^−3^), then steadily increased to a peak in September (78 mg C m^−3^) before decreasing in autumn back to winter values (12 mg C m^−3^; Figure 2a). Analysis of the 16S rRNA gene amplicons from pre‐filtered seawater (Figure 3a) showed that among heterotrophic bacterioplankton, three phyla (Actinobacteriota, Bacteroidota and Proteobacteria) dominated this ecosystem, representing up to 96% of sequence reads during spring. In contrast, Cyanobacteriota had a highly dynamic pattern with a low relative abundance at the beginning of spring (~3% of reads) increasing to a maximum (~38% of reads) by late spring. Their representation fell to ~20% of reads during summer and further decreased to ~12% of reads in autumn (Figure 3a). These shifts emphasize significant seasonal shifts in the bacterioplankton community composition as supported by NMDS analysis (Figure 4) and adonis test results (*R* ^2^ = 0.57, *p* value = 0.001).

The phytoplankton biomass showed considerable temporal variability, with distinct peaks in different size classes and taxonomic groups through the year (Figure 2). In particular, picophytoplankton (< 3 μm) reached a first biomass maximum in June and then again in August (~72 mg C m^−3^). Larger phytoplankton (> 3 μm) showed highest biomass in spring, peaking at 88 mg C m^−3^ in April (Figure 2a,b). Distinct transitions in planktonic biomass among eukaryotic taxa were observed. During the spring bloom, Dinophyceae made up to 80% of total phytoplankton biomass. From spring to summer, biomass shifted from Dinophyceae to Litostomatea and then to Cyanophyceae. By autumn, Diatomophyceae showed a substantial increase in biomass (Figure 2b). Molecular evidence in the form of 18S rRNA gene sequencing highlighted the dominance of dinoflagellates in spring (up to 98% of sequence reads) and showed persistently high proportions year‐round (Figure 3b)– substantially higher than the proportions observed by microscopy (Figure 2b). Molecular identification of diatoms using 18S rRNA gene sequencing proved inconclusive due to insufficient taxonomic resolution. An NMDS analysis and adonis test on the 18S rRNA gene data revealed a strong seasonal effect on eukaryotic community composition (*R* ^2^ = 0.32, *p* value = 0.001; Figure 4) underscoring that temporal environmental variations significantly shape microbial community structure.

### 3.3 Overview of Phosphorus Strategies in the Planktonic‐Microbial Community

**FIGURE 5:**
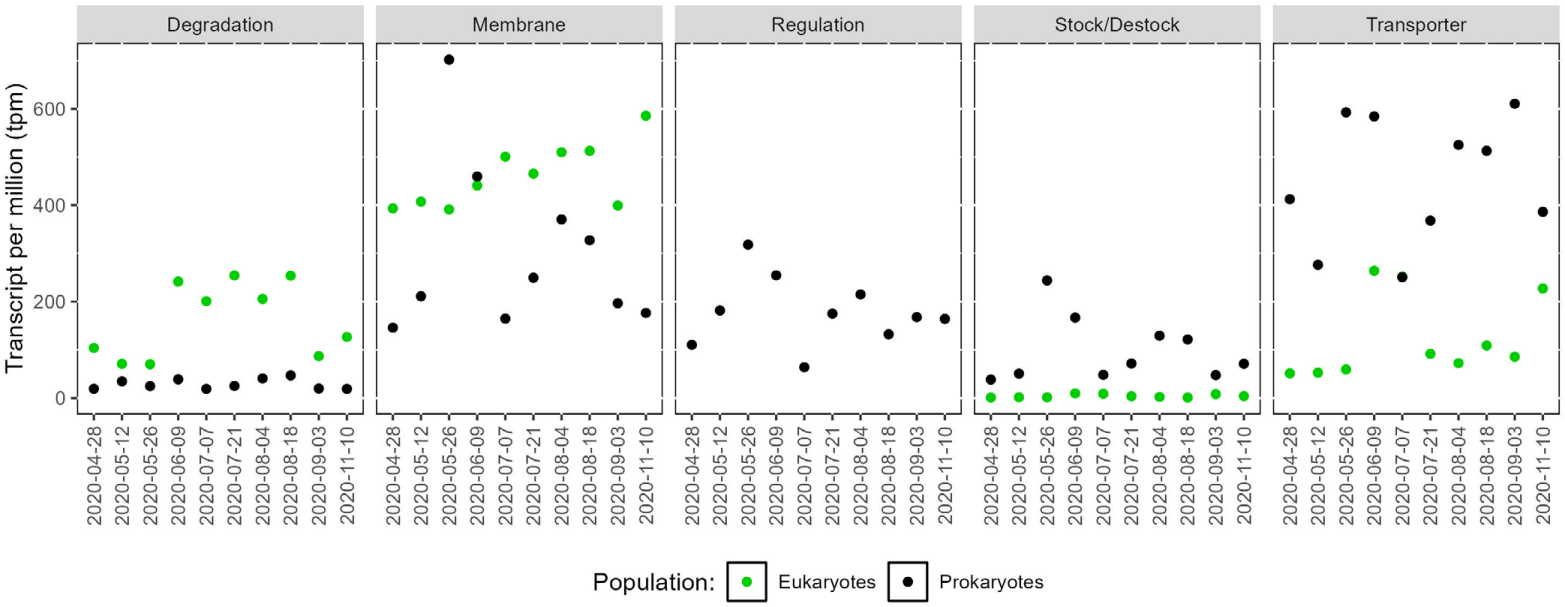
Changes in relative expression levels (TPM) for all P‐cycle functional/quantified genes grouped into five P‐metabolism categories, for free‐living prokaryotes (black) and eukaryotes (green) communities. Only the dates for which both metatranscriptomes were available are shown.

Profiling of the relative gene expression of the microbial communities revealed insights into different P metabolism‐associated gene categories, highlighting pronounced differences between eukaryotic and free‐living prokaryotic expression patterns (Figure 5). Among the total P‐related gene expression, membrane remodelling‐related genes were most frequently detected (41.5% of P‐related gene expression), primarily in the eukaryotic metatranscriptome, indicating a critical role in maintaining cellular integrity. Transporter genes followed at 31.5%, predominantly expressed in free‐living prokaryotes, highlighting their function in nutrient acquisition (Figure 5). Degradation‐related genes, largely associated with eukaryotes, contributed 10.7%, reflecting the breaking down of organic P compounds—a notable contribution that underscores the active involvement of eukaryotic phytoplankton in organic P processing within this system. Stock/destock genes accounted for 5.7% and were more prevalent in free‐living prokaryotes, underscoring their role in P storage regulation. Finally, genes related to the regulation of P metabolism accounted for 10.6% of P gene expression and were exclusively found in the free‐living prokaryotic metatranscriptomes.

### 3.4 Phosphorus Strategies in Free‐Living Prokaryotes

**FIGURE 6:**
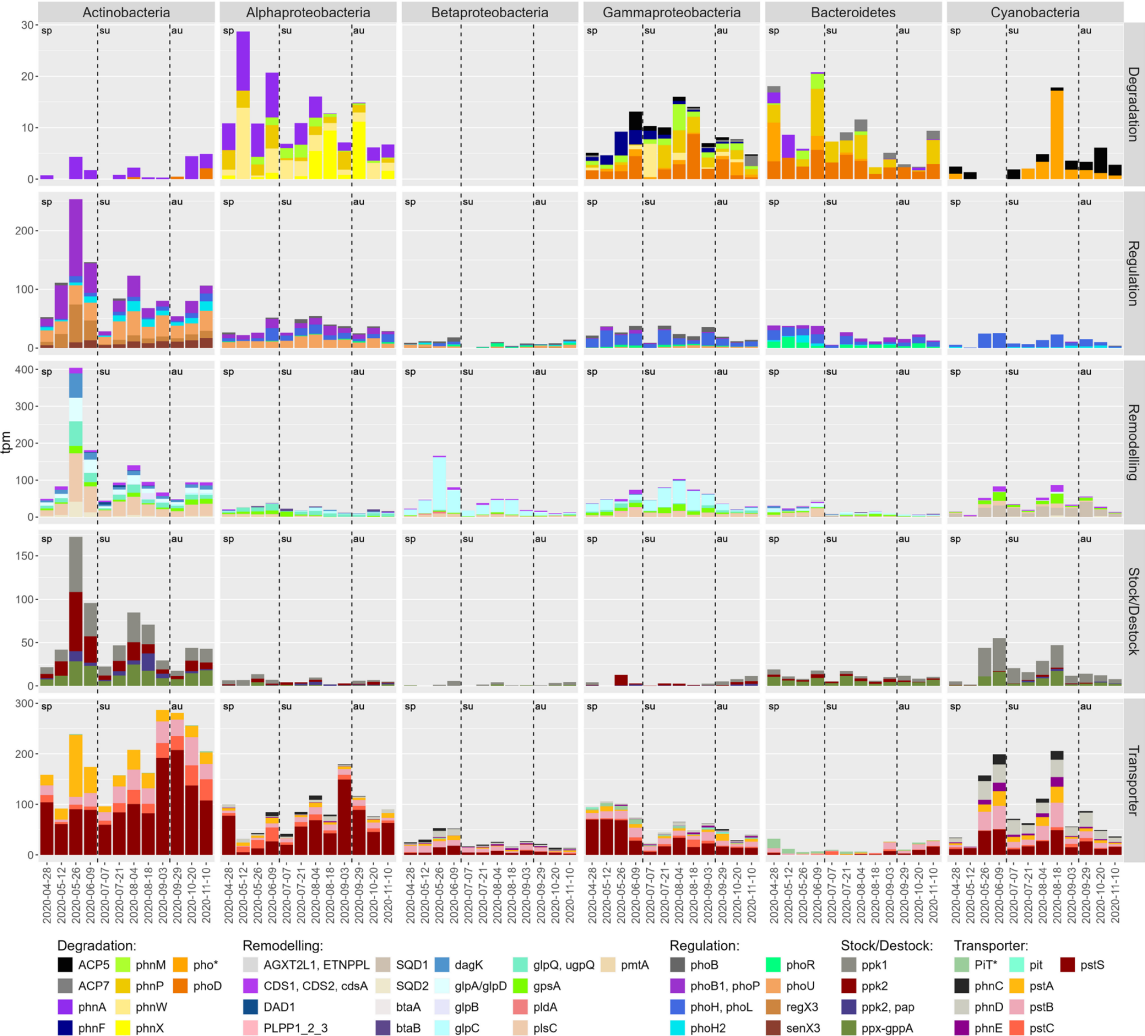
Free‐living prokaryotic gene expression related to the P‐cycle. The 48 functional genes were grouped per phylum across the five P acquisition strategies. In this dataset, Cyanobacteria sequences are representative of picocyanobacterial due to 3 μm pre‐filtration. Please note that the *y*‐axis is different for each gene category. The dashed bars delimit the seasons: au: autumn; sp: spring; su: summer.

The most highly represented phyla in the P‐related gene expression were Actinobacteria, Proteobacteria (Alpha‐, Beta‐ and Gamma‐) and Cyanobacteria (Figure 6). P transporter genes showed the highest levels of relative expression, with the *pstS* gene (part of the PstSCAB phosphate transporter) being highly abundant across all free‐living prokaryotic groups except Bacteroidetes. The other genes coding for this transporter (*pstCAB*) were detected in all groups, although at lower relative abundances.

Actinobacteria demonstrated the highest relative gene expression in all P‐metabolism categories, except degradation, highlighting the importance of this taxonomic group (Figure 6). Regulatory processes were dominated by the *pho* operon with *phoB* (*phoB1/phoP*) and *phoU* contributing 33% and 27% of regulatory transcripts, respectively. Genes coding for *regX3/senX3*, involved in responding to changes in phosphate availability, represented 25% of the tpm in this category. These genes are from the same OmpR family as *phoR/phoB* and regulate responses to phosphate starvation (mostly described in *Mycobacterium*; Glover et al. 2007). In the stock/destock category, polyphosphate kinase analogues (*ppk1*, *ppk2* and *ppk2/pap*), involved in polyphosphate synthesis and degradation (Lin et al. 2016; Sanz‐Luque et al. 2020), accounted for 74% of the tpms. The other gene expressed in this category was *ppx‐gppA* (exopolyphosphatase), commonly used to degrade polyphosphate (Lin et al. 2016; Sanz‐Luque et al. 2020). The membrane remodelling category was dominated by *plsC* (acyltransferase, 34% of category tpm), together with *glpA/glpD* and *glpQ/ugpQ* at 15% each and involved in the conversion of sn‐glycerol‐3P into glycerone phosphate.

Within the Proteobacteria, genes related to degradation of external sources of P showed different patterns in each proteobacterial subclass (Figure 6). Alphaproteobacteria were focused on the degradation of phosphonates, through the expressing of the *phn* genes (Clark et al. 1998). Their most highly expressed membrane category gene was *glpQ*/*ugpQ* (a phosphodiesterase), contributing 35% of total membrane metabolism‐related transcripts. This gene is involved in the addition or removal of a glycerol‐3‐phosphate either to choline or ethanolamine. Gammaproteobacteria expressed genes coding for both alkaline (*phoD*) and acidic (*ACP5, ACP7*) phosphatases with similar levels between phosphatase and phosphonase‐related genes (Figure 6). Additionally, high expression of *gplC* (a glycerol dehydrogenase) accounted for 47% of membrane remodelling transcripts, emphasizing their role in glycerophospholipid and glycerolipid pathways. Betaproteobacteria, while lacking degradation‐associated genes, demonstrated comparable membrane‐associated gene expression levels to Gammaproteobacteria. Notably, *glp*C dominated membrane remodelling activity, contributing 75% of membrane category transcripts, suggesting specific adaptation strategies in Betaproteobacteria despite the absence of degradation capabilities. Despite a relative abundance similar to Proteobacteria (Figure 3a), Bacteroidota displayed markedly few P metabolism‐related genes, with only 912 tpm compared to over 4800 tpm for Proteobacteria (Figure 6).

Gene expression in Cyanobacteria, dominated by Synechococcales at 90% of cyanobacterial transcripts due to the pre‐filtering step, revealed a highly specialized metabolic focus with fewer than seven genes expressed across functional categories (Figure 6). Degradation‐related genes were limited to alkaline phosphatase (pho*, 63% of degradation category tpm) and an acid phosphatase (*ACP5*, 37%). Membrane remodelling was characterized by high expression of *SQD*, involved in synthesizing P free lipids (Mühlroth et al. 2017), and *gpsA* (dehydrogenase), which accounted for 21% of gene category tpm, by converting sn‐glycerol‐3P into glycerone phosphate and *CDS1/CDS2/cdsA* (14% total category tpm) involved in the phosphatidylethanolamine synthesis. Additionally, high expression of phosphonate transporters (*phnC*, *phnD* and *phnE*) was observed, up to 37% of gene category tpm, with *phnD* being the most expressed (254 total tpm). However, unlike the Alphaproteobacteria, no expression of phosphonate degradation genes was detected.

**FIGURE 7:**
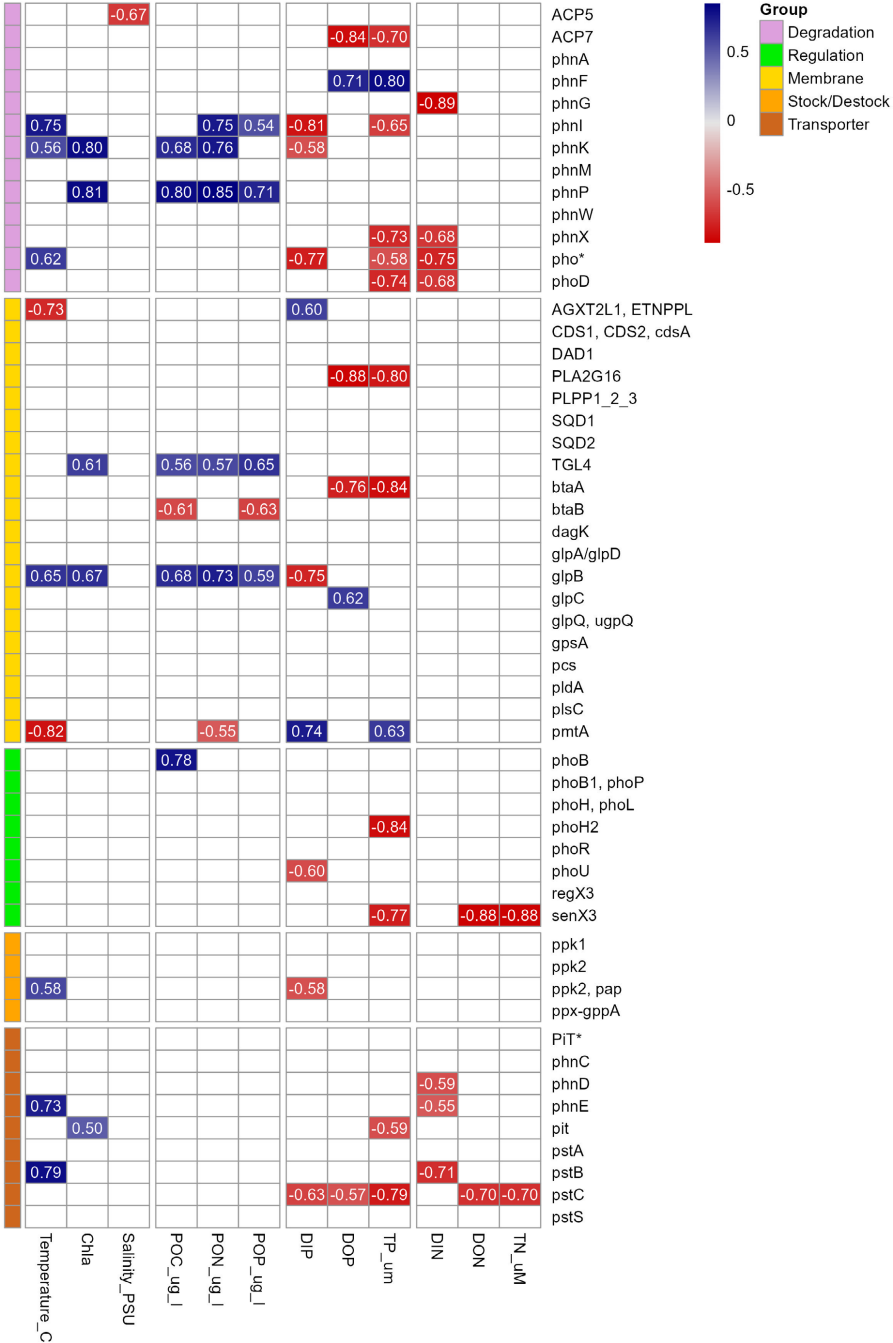
Spearman rank correlation matrix for environmental variables and select genes within the five P acquisition groups. Values in red are negatively correlated, values in blue are positively correlated, all values are significant at the *p* < 0.05 level. A blank square indicates non‐significant correlation. Includes 12 samples collected at the LMO between February 2020 and April 2021.

Phosphorus metabolism‐related gene expression in free‐living prokaryotes showed significant seasonality, peaking during the summer (adonis, *F* = 3.66, *p* value = 0.004). Phosphorus metabolism‐related gene expression was positively correlated with temperature, negatively correlated with DIP, DIN and TP (Spearman's correlations, Figure 7). Most genes across different functional groups were positively correlated, suggesting concurrent activity in various metabolic pathways (data not shown).

### 3.5 Phosphorus Strategies in Eukaryotic Phytoplankton

**FIGURE 8:**
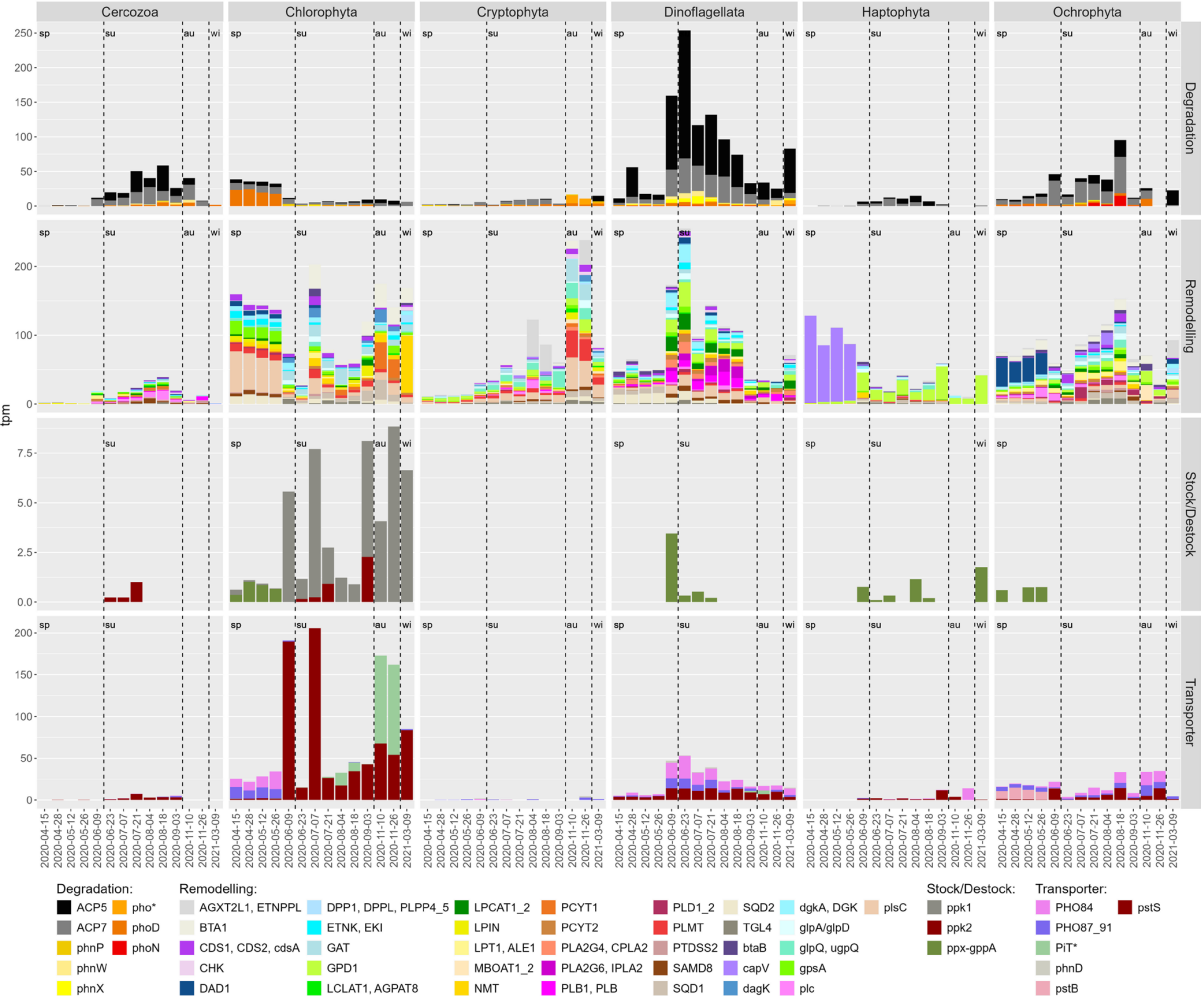
Eukaryotic gene expression related to the P‐cycle. The 53 functional genes were grouped per phylum across the four P acquisition strategies. Please note that the *y*‐axis is different for each gene category. The dashed bars delimit the seasons: au: autumn; sp: spring; su: summer; wi: winter.

Membrane remodelling was identified as the most predominant P‐related gene expression category across the eukaryotic phytoplankton (Figure 8). This category demonstrated remarkable genetic diversity (> 25 different genes), while other categories exhibited fewer genes (< 7). Within the degradation category, acid phosphatases (*ACP5* and *ACP7*) were dominant with 90% of category tpm, highlighting their important role across phytoplankton groups. Similarly, *PHO*84 and *pst*S (39% each of gene category tpm) and *PHO87_91* (18% tpm) emerged as the most expressed genes in the transporter category, underlining the importance of inorganic phosphate transporters. The stock/destock category had the lowest relative expression levels.

Dinoflagellata showed a complex set of highly expressed genes and pathways crucial for membrane remodelling and lipid metabolism. Phospholipase genes, such as *PLB1/PLB, PLA2G4/CPLA2* and *PLA2G6/IPLA2*, play a significant role in producing phosphocholine and phosphatidylethanolamine needed for membrane synthesis and turnover, together accounting for 20% of category tpm (Figure 8). This reversible pathway depends on intermediates like phosphatidylethanolamine and ethanolamine through enzymes *PCYT (*4%) and *ENTK* (3%). Another pathway detected was in the conversion of sn‐glycerol‐3P into glycerone phosphate representing 17% of the detected genes (e.g., *GPD1*, *gpsA* or *glpA/glpD*). Additionally, glycerophospholipid biosynthesis was featured via genes such as *pls*C, or *SAMD8*, and *BTA1*. Genes involved in the formation of 1,2‐diacyl‐sn‐glycerol and 1,2‐diacyl‐sn‐glycerol‐3‐P (as *plsC* or *SAMD8*) represented 14% of the category tpm. These two molecules are central in the glycerophospholipid pathway as they are intermediate in the formation of phosphatidylcholine or phosphatidylcholine and can also be used to form P‐free betaine lipids as diacylglyceryl‐*N,N,N*‐trimethylhomoserine (DGTS). Genes used for the synthesis of betaine lipids as *BTA1* or *btaB* were also detected. In the degradation category, acid phosphatases (*ACP5* and *ACP7*) predominated over alkaline counterparts (*phoD*) or enzymes linked to phosphonate degradation (*PhnX/PhnW*, Figure 8).

Chlorophyta showed notable seasonal variations in the relative expression of genes in the categories for membrane remodelling, transporters and degradation. In spring, the membrane remodelling genes *plsC* (32% of category tpm), *gpsA* (10%) and *SQD2* (7%) dominated, while summer revealed a more balanced expression with *BTA1* (betaine lipid synthase) and *GAT1* (a transferase involved in the formation of glycerone phosphate) each contributing 13%, shifting in autumn to *PCYT1* (20%), *BTA1* (18%) and *SQD1* (12%) of the category tpm each. Most of the genes detected in summer (17 out of 32) represented between 1% and 5% of the total category tpm (Figure 8). Regarding transporters, spring was marked by the dominance of *PHO84* and *PHO87_91* which together accounted for 94% of gene category tpm, but with a low average of 30 tpm. By summer, *pstS* became predominant at 92% (gene category tpm) peaking over 190 tpm, whereas autumn showed a shared dominance between PiT* (low affinity transporter) and *pstS* at 64% and 36%, respectively. The expression of degradation‐related genes was low with an average of 36 tpm during April–May and < 10 tpm the rest of the year (Figure 8). Degradation peaked in spring when acid phosphatases *ACP5* and *ACP7* contributed to 54% of the category expression followed by alkaline phosphatase *phoD* at 42%.

Ochrophyta exhibited a seasonal gene expression dominated by membrane remodelling and degradation processes, with a panel of genes during June–September and a couple of genes peaking in October–November. During spring (April–May), *DAD1* (a phospholipase) dominated with 34% of gene category tpm, highlighting its role in membrane integrity. In contrast, autumn (October–November) showed a more diverse profile with *GPD1* (23%), *MBOAT1_2* (20%), *BTA1* (18%) and *btaB* (12%) driving the remodelling related processes. Degradation activities were mostly driven by acidic phosphatases *ACP5* and *ACP7* accounting for 85% of gene category tpm, while alkaline phosphatase *phoD* and *phoN* contributed 14% of tpm. Haptophyta exhibited the lowest gene expression levels in the transporter and degradation categories, in comparison to other phytoplankton groups. Seasonal shifts in membrane remodelling showcased the dynamic regulation of lipid biosynthesis genes. During spring (April–May), *capV* dominated with 84% of expression in the membrane category, facilitating the formation of essential phospholipids like phosphatidylcholine, ‐ethanolamine and ‐serine. For the rest of the year, however, GPD1 emerged as the leading gene at 67% category tpm, mediating the conversion of sn‐glycerol‐3P to glycerone phosphate.

**FIGURE 9:**
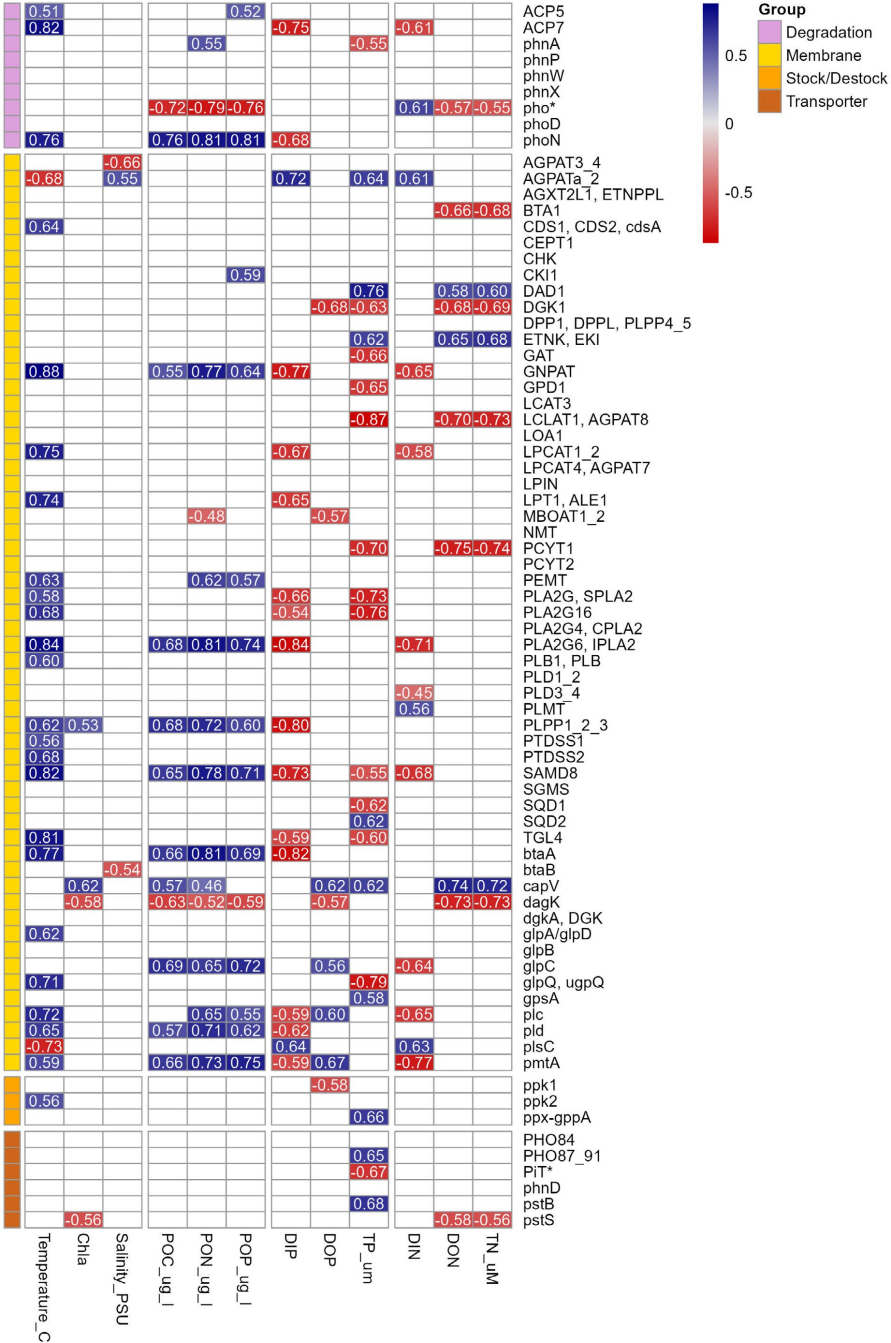
Spearman rank correlation matrix for environmental variables and select genes within the four P acquisition groups. Values in red are negatively correlated, values in blue are positively correlated, all values are significant at the *p* < 0.05 level. A blank square indicates non‐significant correlation. Includes 14 samples collected at the LMO between February 2020 and April 2021.

Complex patterns were observed in the expression of eukaryotic P‐related genes, with distinct seasonal shifts observed in certain classes, like Chlorophyta or Ochrophyta, even if not significantly different statistically (adonis, *F* = 0.89, *p* value = 0.58). Seasonal trends in relative gene expression were linked with variation in environmental conditions, with most genes positively correlating with temperature while exhibiting negative correlations with DIP and DON (Figure 9). In contrast, springtime presented increased relative expression of membrane remodelling genes such as *AGPATa2* and *plsC*. These genes displayed positive correlations with DIP and negative correlations with temperature.

## 4 Discussion

The central Baltic Proper offers a unique setting to investigate P dynamics and acquisition strategies among pelagic communities, as it experiences a seasonal shift from nitrogen (N) limitation in spring to P limitation in summer (Degerholm et al. 2006; Legrand et al. 2015). The sampling period represents a typical year in the Baltic Proper based on environmental parameters and community composition. Throughout the seasons, DOP utilization was observed (Mollica et al. 2025), coinciding with a higher expression of genes coding for phosphatases in certain groups, such as Dinoflagellata and Gammaproteobacteria. Across the microbial‐planktonic community, genes related to membrane remodelling were most abundantly expressed, followed by transporter‐related genes. However, distinct trends were evident among communities, as free‐living prokaryotes mainly expressed transporters and membrane remodelling genes, while eukaryotic communities mainly expressed membrane remodelling genes and degradation genes, highlighting different strategies to cope with P availability.

The bacterial community composition across the year resembled previous records at LMO, with Bacteroidota and Proteobacteria peaking in spring (Lindh et al. 2015; Bunse and Pinhassi 2017; Fridolfsson et al. 2023). However, discrepancies between 16S rRNA gene sequencing and metatranscriptomic data were observed. While DNA data showed similar abundances of Bacteroidota and Proteobacteria, metatranscriptomic data revealed that Bacteroidota had a transcript count five times lower than Proteobacteria. Additionally, 16S rRNA gene sequencing detected only Alphaproteobacteria and Gammaproteobacteria, while Betaproteobacteria were observed in the metatranscriptome, accounting for 20% of all Proteobacteria transcripts, suggesting they may be transcriptionally active but less abundant in the community.

Identification of eukaryotic plankton using 18S rRNA gene amplicon libraries confirmed a dominance of Dinoflagellata during the spring bloom, indicating a shift from diatoms to dinoflagellates supported by microscopic enumeration (Mollica et al. 2025) and long‐term monitoring (Klais et al. 2011). However, Bacillariophyceae were absent from amplicon libraries although they were observed microscopically, suggesting that molecular methods alone may not provide a complete picture of eukaryotic phytoplankton composition. Therefore, integrating traditional microscopy with molecular tools is recommended for better characterization of community dynamics (Santi et al. 2021; Andersson et al. 2023; Latz et al. 2024).

### 4.1 Bacterial Acquisition Strategies: From External to Internal P Sources

The phosphate scavenging through high‐affinity phosphate transporters (pstSCAB) is widely recognized as a primary P acquisition mechanism across marine and freshwater environments (Martiny et al. 2006; Gardner and McCleary 2019). In our dataset, it was the predominant strategy among free‐living bacterioplankton in the Baltic Sea proper, particularly in summer when P availability was the lowest. The dominance of *pst*S expression in our dataset mirrors the global pattern observed in oligotrophic open‐ocean environments, where low DIP concentrations select for bacteria expressing high‐affinity phosphate transporters (Karl 2014; Sunagawa et al. 2015; Guidi et al. 2016; Martiny et al. 2019). However, unlike in oligotrophic oceanic gyres, where *Prochlorococcus* and *Pelagibacter* are the main *pst*S‐expressing taxa (Coleman and Chisholm 2010; Alonso‐Sáez et al. 2023), our dataset highlights that Proteobacteria (Gammaproteobacteria) and Actinobacteria were the dominant *pst*S‐expressing groups. This difference reflects distinct microbial biogeography and environmental constraints of the Baltic Sea, a brackish, low‐salinity and turbid system. Because these conditions select for different taxonomic assemblages than those found in oligotrophic oceanic environments, it is expected that the identity of pstS‐expressing taxa diverges accordingly. Actinobacteria are among the most abundant bacteria in fresh‐ and brackish waters (Bunse and Pinhassi 2017), and have a high capacity for nutrient uptake, including P (Ghylin et al. 2014). Investigations into microbial communities across river‐to‐ocean gradients have shown that genes involved in P‐transport are present and expressed in various environments (Fortunato and Crump 2015). While these studies do not specifically attribute *pstS* expression to Actinobacteria, our dataset revealed that Actinobacteria exhibit strong *pstS* expression, consistent with their major role in high‐affinity phosphate uptake in the Baltic Sea. Thus, while *pstSCAB*‐mediated phosphate uptake is a globally conserved strategy, the dominant taxa responsible vary by ecosystem, reflecting environmental conditions and nutrient availability. In coastal and estuarine systems (Moore et al. 2005; Dyhrman et al. 2007), phosphate acquisition fluctuates seasonally, as observed in the Baltic Sea. High‐affinity transporters (*pho*) are upregulated under low P conditions, but phosphonate degradation genes (phn operon) are also prominent in the global ocean, suggesting the use of an alternative P source to DIP (Lockwood et al. 2022). Organic P hydrolysis through alkaline phosphatases (*phoX, phoD*) and phosphonatases (*phn* genes) is an important pathway for P‐acquisition in some marine environments (Dyhrman et al. 2007; Sebastian and Ammerman 2009). However, our dataset shows a significantly lower expression of DOP degradation genes (*phoX*) for all seasons compared to phosphate transport genes, suggesting a greater reliance on direct phosphate uptake rather than DOP hydrolysis in our study region.

Polyphosphate (polyP) metabolism provides an alternative P‐source under limitation (Sanz‐Luque et al. 2020). In our dataset, Actinobacteria exhibited active polyP metabolism, especially under P‐depleted conditions. This contrasts with global observations in the oligotrophic ocean where polyP accumulation is primarily associated with *Trichodesmium* and heterotrophic bacteria, particularly Alphaproteobacteria (Martin et al. 2014). The TARA Oceans metagenomic survey found that polyP metabolism genes were enriched in certain coastal and mesotrophic sites, particularly in Cyanobacteria and Proteobacteria (Sunagawa et al. 2015). However, our dataset suggests that Actinobacteria play a unique role in polyphosphate utilization in the Baltic Sea, a feature not widely reported in global oceanic surveys. Polyphosphate accumulation is common in low‐salinity environments, with a pronounced role for Betaproteobacteria (Hesselmann et al. 1999). In P‐rich upwelling zones, studies have shown that polyP degradation lags behind dissolved inorganic phosphate uptake, indicating polyP storage during transient P abundance (Martin and Van Mooy 2013). In our dataset, polyP formation and degradation genes (*ppk1, ppk2*) and degradation‐only gene (*ppx*) (Gardner and McCleary 2019; Solovchenko et al. 2019) were detected, particularly in Actinobacteria, with expression correlating with phosphate starvation response regulators (*phoH, phoB1/phoP, regX3*) (Kim et al. 1993; Sola‐Landa et al. 2003; Glover et al. 2007; Gardner and McCleary 2019). This suggests that polyphosphate degradation, rather than accumulation (implying continuous polyP utilization rather than storage), serves as a key strategy for Actinobacteria to maintain P homeostasis. Overall, our findings highlight Actinobacteria as key polyP utilizers, suggesting taxonomic variability in polyP cycling based on environmental conditions.

Membrane remodelling, where P‐based lipids are replaced with sulfur‐ or nitrogen‐based alternatives, is a well‐documented adaptation to P limitation (Van Mooy et al. 2009; Mühlroth et al. 2017). The TARA Oceans dataset reported widespread lipid remodelling genes in heterotrophic bacteria, particularly in Pelagibacterales (Alphaproteobacteria) and Cyanobacteria from P‐limited regions (Sebastián et al. 2016). Our study confirms this trend but with key differences in dominant taxa, as genes associated with phospholipid degradation and substitution (*plcP, glpC, gpsA, plsC*) were widely expressed in Actinobacteria, Gammaproteobacteria and Cyanobacteria. Cyanobacteria, picocyanobacteria in our dataset due to filtration setting, showed high expression of sulfolipid biosynthesis genes (*SQD1, SQD2*), consistent with previous observations from the open ocean (Van Mooy et al. 2009). In oligotrophic open oceans, Pelagibacterales (SAR11) and *Prochlorococcus* are known to substitute phospholipids with sulfolipids under P‐limitation (Carini et al. 2015). Membrane remodelling has also been documented in heterotrophic bacteria, with glycosyltransferase and *plcP* as important genes (Carini et al. 2015; Sebastián et al. 2016). It appears that *plcP* is well distributed and has been identified in several Alphaproteobacteria, Gammaproteobacteria, or Verrucomicrobia (Sebastián et al. 2016). Freshwater systems frequently experience P limitation, but the extent of lipid remodelling in response to P scarcity appears to be less pronounced compared to marine systems. A study on the freshwater cyanobacterium *Microcystis aeruginosa* found that while sulfolipid substitution occurs under P limitation, the degree of lipid remodelling is less extensive than that observed in marine species (Martin et al. 2023). Our data indicate that Actinobacteria and Gammaproteobacteria are the primary taxa expressing lipid remodelling genes in brackish waters, reflecting distinct microbial communities between these environments. Overall, while lipid remodelling is a widely distributed response to P stress, our dataset highlights ecosystem‐specific differences, particularly in the dominant bacterial taxa employing this strategy.

### 4.2 Eukaryotic Phytoplankton: Membrane Remodelling as Main P‐Saving Response

Substantial research has explored P‐acquisition mechanisms in oceanic environments across both prokaryotic and eukaryotic organisms, though with notable disparities in focus and depth between the two groups. While P‐related gene expression in marine bacterioplankton and picocyanobacteria is well‐documented, revealing crucial adaptive mechanisms, fewer studies have investigated eukaryotic phytoplankton (White 2009; Sunagawa et al. 2015; Carradec et al. 2018; Groussman et al. 2024). To address this gap, our study examines key genetic pathways related to membrane remodelling in eukaryotic phytoplankton, observing extensive diversity across taxa during seasonal shifts in the Baltic Proper. This genetic diversity underscores a significant adaptive potential to fluctuating P levels and aligns with findings in dynamic coastal environments (Lin et al. 2016). Membrane remodelling emerged as the most expressed process across all phytoplankton groups but was executed via taxon‐specific strategies. For example, diatoms like *Phaeodactylum tricornutum* can replace phospholipids by betaine lipids under P‐limitation conditions (Cañavate et al. 2017), while Chlorophyta such as *Chlamydomonas reinhardtii* substitute phosphatidylglycerol by sulfoquinovosyldiacylglycerol to maintain thylakoid membrane integrity (Iwai et al. 2014). Our dataset identified two primary lipid substitution pathways: one involving betaine lipid synthesis driven by highly expressed genes like *BTA1* and *btaB*, constituting approximately 12% and 11% of total membrane‐remodelling tpms for Ochrophyta and Chlorophyta respectively, and another focusing on sulfolipid biosynthesis via *SQD1* and *SQD2* expression, predominantly active in Dinoflagellata and Chlorophyta (Güler et al. 2000; Huang et al. 2023). Dinoflagellata here demonstrated potential phospholipid membrane remodelling as an adaptive strategy to sustain growth in P‐depleted coastal waters by conserving P through phospholipid‐to‐non‐phospholipid conversions—a finding consistent with observations in *Karenia mikimotoi* by Huang et al. (2023). These findings suggest that phospholipid membrane remodelling serves as an essential adaptive P‐saving response allowing diverse phytoplankton taxa to reduce phosphate demand while recycling Pi under P‐limited conditions.

Polyphosphates are traditionally regarded as a significant internal P‐reservoir for cells; however, in this study, their role appears limited due to low gene expression linked to polyphosphate metabolism. For example, *VTC4*, which codes for the vacuolar transporter chaperone and is crucial for polyphosphate synthesis in eukaryotes (Lin et al. 2016; Sanz‐Luque et al. 2020) was undetected. Similarly, the low expression of polyphosphate kinase genes (*ppk1* and *ppk2*) further restricts our understanding of polyphosphate utilization. To acquire external phosphate (Pi), two primary strategies emerged: reliance on inorganic P transporters or degradation of DOP. Chlorophyta exhibited increased expression of transporters during summer and autumn, with a notable shift based on ambient dissolved inorganic phosphorus (DIP) concentrations. Under DIP scarcity in summer (< 0.3 μM), high‐affinity transporter pstS was dominant (Lin et al. 2016), whereas seasons with higher DIP levels favoured the expression of low‐affinity transporters like *pho87_91* or *PiT* (Ghillebert et al. 2011; Lin et al. 2016). Conversely, Dinoflagellata relied more heavily on DOP degradation during low DIP conditions, as evidenced by a tenfold increase in related gene expressions such as *ACP5* and *ACP7* during summer compared to spring; a trend persisting into autumn despite reduced bacterial biomass potentially contributing less to DOP turnover (Mollica et al. 2025). This dataset suggests that phytoplankton exhibit diverse adaptive mechanisms to cope with Pi limitation: some groups emphasize Pi uptake via transporters while others degrade DOP or remodel membranes to recycle Pi efficiently. This highlights the complexity of survival strategies employed by phytoplankton under varying P availability while challenging traditional assumptions about polyphosphates' centrality as cellular P reserves.

### 4.3 Complementarity and Adaptability of the Microbial Planktonic Communities

The expression of P‐related genes in both eukaryotic phytoplankton and free‐living bacteria was predominantly observed during the summer and correlated with high temperatures and low nutrient conditions. A significant positive correlation was identified among most P‐related genes, indicating a shared genetic response to environmental cues. While not all eukaryotic P‐related genes exhibited seasonality, specific classes displayed clear temporal shifts, reflecting their adaptive strategies to changing conditions. Notably, differences emerged between the adaptive mechanisms of free‐living bacteria and eukaryotic phytoplankton. In free‐living bacteria, the pstSCAB genes—high‐affinity Pi transporters typically expressed under low Pi availability (Gardner and McCleary 2019)—were overexpressed in summer, suggesting that these organisms prioritize scavenging extracellular Pi sources. Actinobacteria appeared unique within this group due to the more prominent role of polyphosphate metabolism compared to other bacteria. For eukaryotes, membrane remodelling was a primary strategy across all classes for coping with low Pi conditions. Additionally, the number of genes was significantly higher in the eukaryotic metatranscriptome (59 genes) than in the free‐living prokaryotic one (21 genes), reflecting more diverse approaches. In addition, secondary strategies varied by eukaryote group; where, for example, Chlorophyta relied on extracellular P scavenging through specialized transporters and Dinoflagellata emphasized degradation of DOP. Together, these findings highlight distinct yet complementary adaptive responses between free‐living bacteria and eukaryotic phytoplankton to overcome P limitation in their environments. These seasonal strategy shifts would create windows of opportunity favouring different taxa, thus indicating that P‐pools dynamic drive both gene expression and community turnover in the Baltic Sea microbial planktonic community.

## 5 Conclusions

Our study highlights both conserved and ecosystem‐specific P strategies in Baltic Sea free‐living prokaryotic and eukaryotic plankton. For both free‐living bacteria and eukaryotic phytoplankton, P‐related genes were mainly expressed in summer and their expression correlated with high temperature and low nutrient conditions. The heterotrophic bacteria predominantly relied on phosphate scavenging (high‐affinity phosphate uptake) and Actinobacteria exhibited a unique reliance on polyphosphate metabolism. Picocyanobacteria relied mostly on transporters and remodelling of the membrane. Eukaryotic phytoplankton, in contrast, primarily utilized membrane remodelling to conserve P, with secondary strategies varying by group reflecting P availability. Polyphosphate metabolism varies across ecosystems, with Actinobacteria driving this process in the Baltic Sea, in contrast to Cyanobacteria in open oceans. Unexpectedly, our eukaryotic phytoplankton dataset showed low expression of polyphosphate storage genes, unlike in marine and freshwater systems. Membrane (lipid) remodelling is a widespread P conservation strategy where Gammaproteobacteria and Actinobacteria are key remodellers in the Baltic Sea. Among phytoplankton, Dinoflagellata, Ochrophyta and Chlorophyta favour betaine and sulfolipid substitutions. DOP degradation appears less prominent in Baltic Sea bacteria compared to global datasets, but Dinoflagellata play a key role in DOP hydrolysis. These findings emphasize how salinity, seasonal P limitation and microbial composition shape P strategies. While high‐affinity transport and membrane lipid remodelling are conserved, the dominant taxa and reliance on polyphosphate metabolism or DOP degradation appear ecosystem‐dependent. Even though DIP remained above detection limits, rapid biological uptake and strong competition in the Baltic Proper indicate that phosphate supply rate, rather than bulk concentration, can still restrict growth. Our transcriptomic data of high‐affinity transport and P‐scavenging pathways indicate internal P stress, showing that cells experience functional limitation despite measurable DIP. Thus, low DIP can meaningfully constrain microbial growth and shape seasonal community dynamics. This study reinforces the need for regional analyses to complement global omics surveys and refine our understanding of the microbial contribution to P cycling.

## Author Contributions

**M.T.:** study conception, data acquisition, data analysis, data processing. **F.H.:** data process, manuscript contribution. **L.E.:** study conception, manuscript contribution. **L.D.:** data process, data analysis, manuscript contribution. **P.J.:** manuscript contribution, data analysis. **L.C.:** study conception, data analysis, manuscript contribution.

## Funding

This project was supported by the Swedish Research Council FORMAS Strong Research environment EcoChange to CL and JP, and by the Linnaeus University Centre for Ecology and Evolution in Microbial model Systems (EEMiS). The authors acknowledge support from the National Genomics Infrastructure in Genomics Production Stockholm funded by Science for Life Laboratory, the Knut and Alice Wallenberg Foundation and the Swedish Research Council. ‘The computations handling were enabled by resources in project [NAISS 2023‐22‐1153] provided by the National Academic Infrastructure for Supercomputing in Sweden (NAISS) at UPPMAX, funded by the Swedish Research Council through grant agreement no. 2022‐06725.

## Ethics Statement

The authors have nothing to report.

## Conflicts of Interest

The authors declare no conflicts of interest.

## Supporting information


**Table S1:** Details of primers and PCR protocols.
**Table S2:** Denoised statistic after DADA2 for 16 and 18S region, in yellow samples that have not been included in the analysis due to low number of reads.
**Figure S1:** Glycero‐ and glycerophospholipids pathways (KEGG pathways maps). The numbers in pink indicate the location of the genes used in this study.

## Data Availability

The metagenomic data used in this study have been deposited in SRA under the accession number PRJNA123455 (https://dataview.ncbi.nlm.nih.gov/object/PRJNA1234559?reviewer=3dv7ffm2t8v4f098ut5m6d0qbc). The metatranscriptomic data used in this study have been deposited in SRA under the accession number PRJNA1290104 (https://dataview.ncbi.nlm.nih.gov/object/PRJNA1290104?reviewer=s7t245c5sur64mb566kcj2qcr1). Other data that support the findings of this study are available from the corresponding author upon reasonable request.
