# Correction to “Phosphorus Acquisition Strategies Among Phytoplankton and Free‐Living Bacterial Communities in the Baltic Proper”

**DOI:** 10.1111/1758-2229.70387

**Published:** 2026-07-17

Mollica, T. , H. Farnelid , E. Lindehoff , D. Lundin , J. Pinhassi , and C. Legrand . 2026. “Phosphorus Acquisition Strategies Among Phytoplankton and Free‐Living Bacterial Communities in the Baltic Proper.” Environmental Microbiology Reports 18, no. 2: e70332. 10.1111/1758-2229.70332.41990829 PMC13086504

The authors’ first names and surnames in the author byline were displayed incorrectly. The author byline should be corrected to: Thomas Mollica, Hanna Farnelid, Elin Lindehoff, Daniel Lundin, Jarone Pinhassi, and Catherine Legrand.

The online version of the article has been corrected.

We apologise for this error.

