# Supplementary material for: Phosphorus Acquisition Strategies Among Phytoplankton and Free‐Living Bacterial Communities in the Baltic Proper
Source: Environ Microbiol Rep. 2026 Apr 16;18(2):e70332. doi: 10.1111/1758-2229.70332 (PMC13086504; doi:10.1111/1758-2229.70332)
Supplement: Supplementary file 1 — Table S1: Details of primers and PCR protocols. Table S2: Denoised statistic after DADA2 for 16 and 18S region, in yellow samples that have not been included in the analysis due to low number of reads. Figure S1: Glycero‐ and glycerophospholipids pathways (KEGG pathways maps). The numbers in pink indicate the location of the genes used in this study. [file EMI4-18-e70332-s001.docx]

# Supplementary Material:

*Supplementary Table 1: Details of primers and PCR protocols*

|  | Primers F | Primers R | PCR protocol | PCR 2 protocol |
| --- | --- | --- | --- | --- |
| 16S | 341F CCTACGGGNGGCWGCAG  (Herlemann *et al.*, 2011) | 805R GACTACHVGGGTATCTAATCC  (Herlemann *et al.*, 2011) | (1*[98⁰C *30s], 20*[98⁰C, 10s; 58⁰C, 30s; 72⁰C, 15s], 1*[72⁰, 2 min]) | (1*[98⁰C *30s], 12*[98⁰C, 10s; 62⁰C, 30s; 72⁰C, 5s], 1*[72⁰, 2 min]) |
| 18S | 454F CCAGCASCYGCGGTAATTCC  (Stoeck *et al.*, 2010) | V4RB ACTTTCGTTCTTGATYRR  (Balzano, Abs and Leterme, 2015) | (1*[98⁰C *30s], 20*[98⁰C, 10s; 53⁰C, 30s; 72⁰C, 15s], 1*[72⁰, 2 min]) | 1*[98⁰C *30s], 12*[98⁰C, 10s; 62⁰C, 30s; 72⁰C, 5s], 1*[72⁰, 2 min]) |

*Supplementary Table 2: Denoised statistic after DADA2 for 16 and 18S region, in yellow samples that have not been included in the analysis due to low number of reads*

| **Sample** | **Region** | **Input** | **Filtered** | **DenoisedF** | **DenoisedR** | **Merged** | **Non-chimeric** |
| --- | --- | --- | --- | --- | --- | --- | --- |
| StoP 1 | 16S | 245506 | 228792 | 215054 | 223860 | 188451 | 152613 |
| StoP 2 | 16S | 179638 | 166737 | 153501 | 161783 | 131683 | 104365 |
| StoP 3 | 16S | 142134 | 132557 | 120508 | 127333 | 97349 | 63881 |
| StoP 4 | 16S | 187549 | 174557 | 162614 | 169045 | 127396 | 52874 |
| StoP 5 | 16S | 156269 | 145749 | 137440 | 142252 | 114895 | 64099 |
| StoP 6 | 16S | 237102 | 222145 | 213736 | 218241 | 186338 | 107209 |
| StoP 7 | 16S | 188567 | 176236 | 168786 | 172961 | 147577 | 85406 |
| StoP 8 | 16S | 100722 | 93832 | 86994 | 91269 | 68400 | 36168 |
| StoP 9 | 16S | 124495 | 116236 | 110288 | 113503 | 94885 | 53787 |
| StoP 11 | 16S | 73190 | 68119 | 61205 | 65573 | 47673 | 21150 |
| StoP 12 | 16S | 180364 | 167695 | 154189 | 162511 | 122501 | 49966 |
| StoP 13 | 16S | 320844 | 298077 | 269536 | 288038 | 199269 | 51442 |
| StoP 14 | 16S | 241041 | 145376 | 132771 | 140385 | 102182 | 46879 |
| StoP 15 | 16S | 255534 | 157892 | 148679 | 154223 | 123358 | 71895 |
| StoP 16 | 16S | 194284 | 122973 | 114731 | 119296 | 91013 | 50056 |
| StoP 17 | 16S | 222462 | 141138 | 133908 | 138242 | 112458 | 64426 |
| StoP 18 | 16S | 166518 | 103193 | 95078 | 99519 | 73758 | 35539 |
| StoP 19 | 16S | 190898 | 118699 | 109442 | 114499 | 85869 | 41896 |
| StoP 20 | 16S | 232617 | 147152 | 137358 | 143166 | 111951 | 67830 |
| StoP 21 | 16S | 279318 | 177156 | 162638 | 170996 | 130920 | 81662 |
| StoP 22 | 16S | 233675 | 151000 | 135283 | 142961 | 101897 | 72489 |
| StoP 1 | 18S | 1490 | 223 | 171 | 175 | 39 | 39 |
| StoP 2 | 18S | 1629 | 173 | 140 | 142 | 18 | 14 |
| StoP 3 | 18S | 184029 | 119868 | 118695 | 118968 | 107578 | 81352 |
| StoP 4 | 18S | 264526 | 171805 | 170304 | 170647 | 155465 | 109379 |
| StoP 5 | 18S | 248583 | 172440 | 171110 | 171300 | 153822 | 100474 |
| StoP 6 | 18S | 195754 | 136940 | 135637 | 136491 | 127031 | 108424 |
| StoP 7 | 18S | 228421 | 151487 | 150014 | 150047 | 126716 | 73671 |
| StoP 8 | 18S | 185364 | 113365 | 112567 | 112282 | 103638 | 59417 |
| StoP 9 | 18S | 262437 | 172573 | 170929 | 171327 | 153525 | 110373 |
| StoP 10 | 18S | 308078 | 204220 | 202911 | 203081 | 185324 | 139958 |
| StoP 11 | 18S | 373104 | 232697 | 231036 | 231333 | 213322 | 123722 |
| StoP 12 | 18S | 158805 | 101238 | 99410 | 99862 | 82308 | 66509 |
| StoP 13 | 18S | 321861 | 205340 | 202625 | 202924 | 176517 | 127970 |
| StoP 14 | 18S | 103831 | 65610 | 64197 | 64456 | 52935 | 44839 |
| StoP 15 | 18S | 382802 | 240461 | 237383 | 237907 | 209504 | 140948 |
| StoP 16 | 18S | 358999 | 227621 | 224927 | 225551 | 203349 | 147251 |
| StoP 17 | 18S | 207442 | 134160 | 132013 | 132218 | 112245 | 87147 |
| StoP 18 | 18S | 247537 | 149952 | 148915 | 148980 | 141672 | 80446 |
| StoP 19 | 18S | 171476 | 108923 | 107313 | 107711 | 95048 | 72637 |
| StoP 20 | 18S | 291695 | 181125 | 179897 | 180127 | 171614 | 141573 |
| StoP 21 | 18S | 263411 | 172370 | 170120 | 170775 | 147194 | 110664 |
| StoP 22 | 18S | 279973 | 189154 | 187678 | 187915 | 174163 | 159623 |
| StoP 23 | 18S | 19 | 6 | 1 | 1 | 0 | 0 |
| StoP 24 | 18S | 31 | 18 | 7 | 10 | 7 | 5 |
| StoP 25 | 18S | 16 | 8 | 1 | 1 | 0 | 0 |
| StoP 26 | 18S | 25 | 15 | 1 | 3 | 0 | 0 |


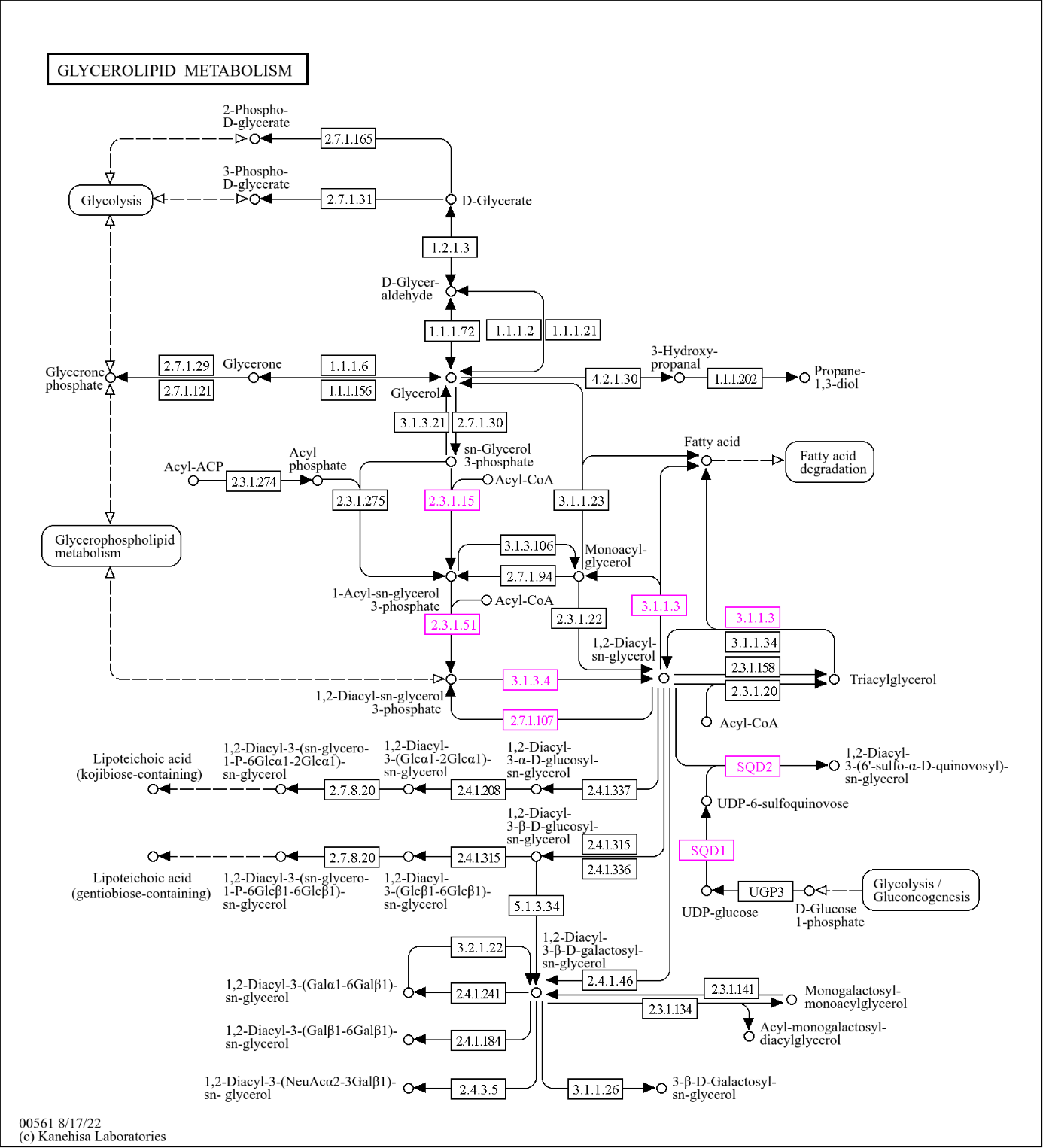


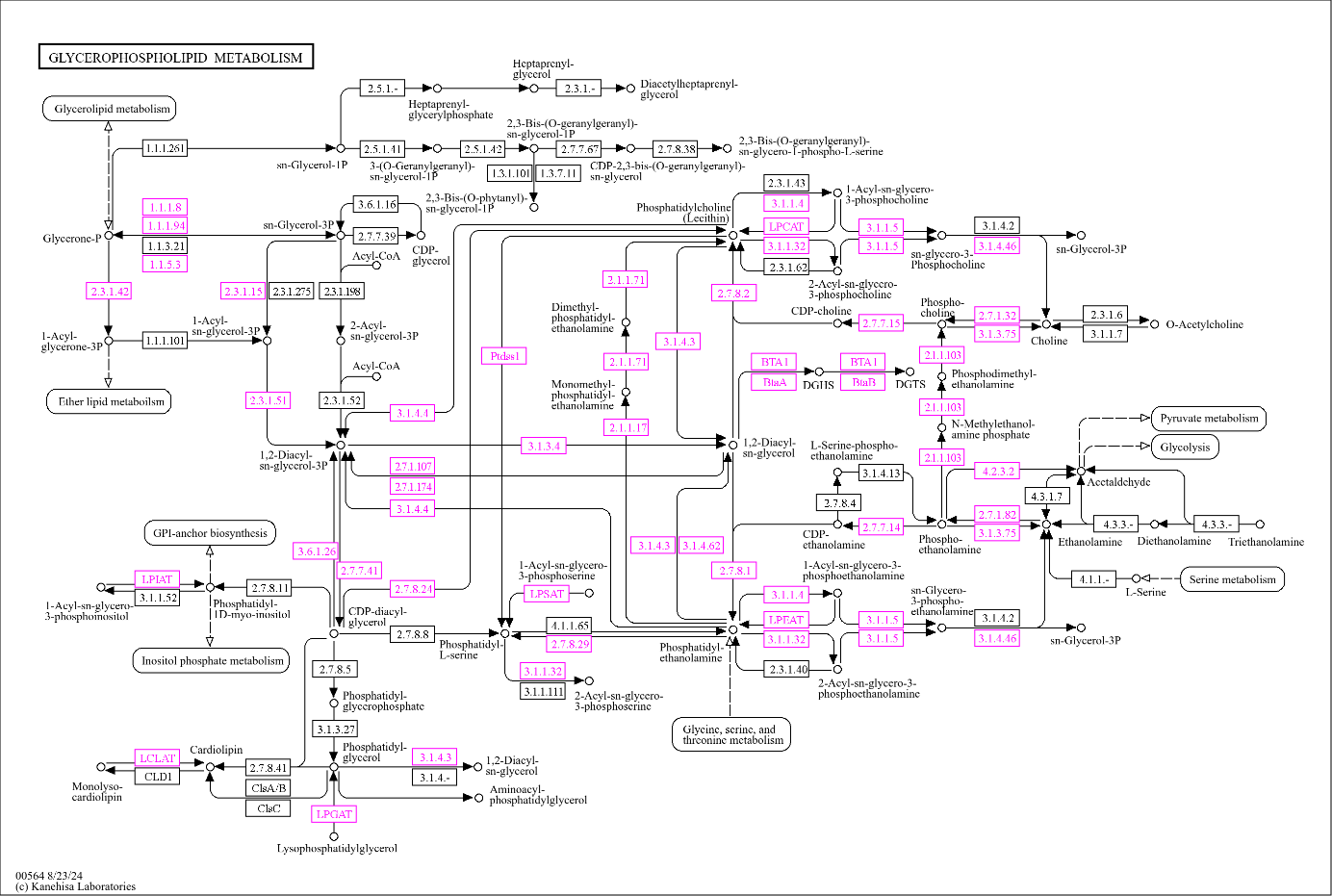


*Supplementary Figure 1: Glycero- and glycerophospholipids pathways (KEGG pathways maps). The numbers in pink indicate the location of the genes used in this study.*
